# Intranasal Delivery of MVA Vector Vaccine Induces Effective Pulmonary Immunity Against SARS-CoV-2 in Rodents

**DOI:** 10.3389/fimmu.2021.772240

**Published:** 2021-11-11

**Authors:** Berislav Bošnjak, Ivan Odak, Joana Barros-Martins, Inga Sandrock, Swantje I. Hammerschmidt, Marc Permanyer, Gwendolyn E. Patzer, Hristo Greorgiev, Rodrigo Gutierrez Jauregui, Alina Tscherne, Jan Hendrik Schwarz, Georgia Kalodimou, George Ssebyatika, Malgorzata Ciurkiewicz, Stefanie Willenzon, Anja Bubke, Jasmin Ristenpart, Christiane Ritter, Tamara Tuchel, Christian Meyer zu Natrup, Dai-Lun Shin, Sabrina Clever, Leonard Limpinsel, Wolfgang Baumgärtner, Thomas Krey, Asisa Volz, Gerd Sutter, Reinhold Förster

**Affiliations:** ^1^ Institute of Immunology, Hannover Medical School, Hannover, Germany; ^2^ Division of Virology, Department of Veterinary Sciences, Ludwig Maximilian University (LMU) Munich, Munich, Germany; ^3^ German Centre for Infection Research (DZIF), Partner Site Munich, Munich, Germany; ^4^ Center of Structural and Cell Biology in Medicine, Institute of Biochemistry, University of Lübeck, Lübeck, Germany; ^5^ Department of Pathology, University of Veterinary Medicine Hannover, Hannover, Germany; ^6^ Institute for Virology, University of Veterinary Medicine Hannover, Hannover, Germany; ^7^ German Centre for Infection Research (DZIF), Partner Site Hamburg-Lübeck-Borstel-Riems, Lübeck, Germany; ^8^ Centre for Structural Systems Biology (CSSB), Hamburg, Germany; ^9^ Institute of Virology, Hannover Medical School, Hannover, Germany; ^10^ Cluster of Excellence RESIST (EXC 2155), Hannover Medical School, Hannover, Germany; ^11^ German Centre for Infection Research (DZIF), Partner Site Hannover, Hannover, Germany

**Keywords:** bronchus-associated lymphoid tissue (BALT), lungs, modified vaccinia virus Ankara (MVA), severe acute respiratory syndrome coronavirus 2 (SARS-CoV-2), spike (S) protein, vaccine, vaccination, respiratory tract

## Abstract

Antigen-specific tissue-resident memory T cells (Trms) and neutralizing IgA antibodies provide the most effective protection of the lungs from viral infections. To induce those essential components of lung immunity against SARS-CoV-2, we tested various immunization protocols involving intranasal delivery of a novel Modified Vaccinia virus Ankara (MVA)-SARS-2-spike vaccine candidate. We show that a single intranasal MVA-SARS-CoV-2-S application in mice strongly induced pulmonary spike-specific CD8^+^ T cells, albeit restricted production of neutralizing antibodies. In prime-boost protocols, intranasal booster vaccine delivery proved to be crucial for a massive expansion of systemic and lung tissue-resident spike-specific CD8^+^ T cells and the development of Th1 - but not Th2 - CD4^+^ T cells. Likewise, very high titers of IgG and IgA anti-spike antibodies were present in serum and broncho-alveolar lavages that possessed high virus neutralization capacities to all current SARS-CoV-2 variants of concern. Importantly, the MVA-SARS-2-spike vaccine applied in intramuscular priming and intranasal boosting treatment regimen completely protected hamsters from developing SARS-CoV-2 lung infection and pathology. Together, these results identify intramuscular priming followed by respiratory tract boosting with MVA-SARS-2-S as a promising approach for the induction of local, respiratory as well as systemic immune responses suited to protect from SARS-CoV-2 infections.

## Introduction

Large vaccination campaigns started at the beginning of 2021 to combat the severe acute respiratory syndrome coronavirus 2 (SARS-CoV-2) pandemic. Although the vaccines are very effective in preventing infection (1–3), they only partially suppressed shedding of the original virus from vaccinated individuals (4), reducing levels of disease transmission of only 50-60% (5). Moreover, SARS-CoV-2 has progressively changed, and several rapidly expanding variants of concern (VoC) emerged: alpha (B.1.1.7), beta (P.1; formerly named B.1.1.28.1), gamma (B.1.351), and most recently delta (B.1.617.2) (6, 7). Unfortunately, current vaccines are less protective against these SARS-CoV-2 VoC (8–13), even when applied in heterologous prime-boost regimens (14). Even more concerning, pre-print data suggest that vaccinees who get infected with delta SARS-CoV-2 variant have high viral loads and, thus, transmit the virus regardless of their vaccination status (15, 16).

In contrast to intramuscular vaccine application, inhalative vaccination induces high levels of antigen-specific IgA antibodies and tissue-resident memory T cells (Trms) in the respiratory tract (17–19). Secretory IgA antibodies cover lung mucosa and protect the lungs by immune exclusion, complexing, and neutralization of invading microorganisms (20). Trms originate from effector T cells that enter the lungs during initial infection and, after pathogen clearance, remain resident within the lung parenchyma and in the airways (21). Upon re-infection, Trms get rapidly activated, secrete cytokines, proliferate, and recruit other leukocytes, thus enabling accelerated clearance of the pathogens (22). Along these lines, respiratory delivery of coronavirus 2019 (COVID-19) vaccine candidates induced robust lung immunity and protected against SARS-CoV-2 infection (23–26). Moreover, inhaled COVID-19 vaccine candidate based on parainfluenza virus type 5 vector efficiently blocked animal-to-animal transmission of the SARS-CoV-2 (27). Currently, there are several COVID-19 vaccine candidates in development for inhaled administration (28) and the latest clinical results proved safety and immunogenicity of aerosolized adenovirus type-5 vector-based COVID-19 vaccine (29).

We already hypothesized that respiratory delivery of a Modified Vaccinia virus Ankara (MVA)-based vaccine would be a favorable approach to combat COVID-19 (30). MVA is a highly attenuated strain of vaccinia virus that is growth adapted to avian cells and replication-deficient in mammalian cells (31, 32). Nevertheless, MVA retained the ability to infect mammalian cells and to induce efficient humoral and cellular immune responses (33) and became a third generation smallpox vaccine, now licensed in Canada and the European Union (34). Moreover, unaffected synthesis of viral proteins in MVA-infected cells enables high levels of recombinant protein production from recombinant MVA (35, 36), making it an advanced viral vector platform for numerous vaccines, including those against coronaviruses (37–40).

Beside systemic immune responses, respiratory delivery of MVA induces strong antigen-specific CD8 immunity and IgA production within lungs (41–43). Moreover, as we have described in detail intranasal application of MVA also leads to development of bronchus-associated lymphoid tissue (BALT) in the lungs of mice (44). BALT are tertiary lymphoid organs composed of B cell follicles surrounded by a para-follicular area rich in T cells and antigen-presenting cells (44–46) and serve as a general priming site for the induction of adaptive immune responses (44).

In this study, we used respiratory delivery of a MVA vector vaccine expressing the spike (S) protein of SARS-CoV-2 (MVA-SARS-2-S) to induce protective lung and systemic immunity in rodents. In line with previous preclinical and clinical data (37, 41, 47–49), respiratory delivery of MVA-SARS-2-S has been shown to be both safe and immunogenic. More importantly, our profound analysis in mice revealed that exclusively immunization protocols involving intranasal boosting induce S-specific Trms and neutralizing IgA antibodies in lung. Particularly, neutralizing antibodies developed by MVA-SARS-2-S were also effective against SARS-CoV-2 VoC and this immunization protocol also efficiently protected hamsters from SARS-CoV-2 infection.

## Material And Methods

### Experimental Animals

C57BL/6N mice were purchased from Charles River and bred and maintained in the Central Animal Facility of Hannover Medical School (Hannover, Germany) under specific pathogen‐free conditions and used for experiments at the age of 7 – 13 weeks. Male syrian hamster (10 week-old, Mesocricetus auratus; breed HsdHan^®^:AURA) were purchased from Envigo RMS Inc. (Indiampolis, United States). Hamsters were maintained under specified pathogen-free conditions, had free access to food and water, and were allowed to adapt to the facilities for at least one week before vaccination experiments were performed. All animal experiments were handled in compliance with the European and national regulations for animal experimentation (European Directive 2010/63/EU; Animal Welfare Acts in Germany) and Animal Welfare Act, approved by the Niedersächsisches Landesamt für Verbraucherschutz und Lebensmittelsicherheit (LAVES) Lower Saxony, Germany).

### Immunization With MVA-Spike

The recombinant virus MVA-SARS-2-S was constructed and characterized in detail as described elsewhere (50). In the present study, a quality controlled vaccine lot was applied serving for preclinical characterization of MVA-SARS-2-S as in preparation of clinical Phase-1 evaluation. For i.n. administration, animals were lightly anaesthetized and indicated plaque forming units (PFU) of MVA-SARS-2-S diluted in 40 μl saline, 15 mM Tris pH7.7, 3% sucrose, 0.005% Tween 80 were applied to the nostrils. For intra muscular immunization, 25 µl of indicated PFU of MVA-SARS-2-S dissolved in 30 mM Tris pH7.7, 6% sucrose, 0.01% Tween 80 were injected into the quadriceps at one of the hind legs.

In mice, we tested MVA-SARS-2 in two different setups: *(i)* prime only (PO), *(ii)* prime – boost (PB) vaccination regiment. In the PO setting, mice received one single i.n. priming dose of MVA-SARS-2-S doses indicated at day 0 and were analyzed at day 11, 24, 35 or 40 post immunizations. For PB immunization protocol, mice primed on day 0 received a second MVA-SARS-2 immunization (boost) on day 24 and were analyzed on day 40 post prime. In all PB experiments, three immunization protocols were compared (1): intranasal (na) priming and boosting (na-na) (2), intramuscular (mu) priming intra nasal boosting (mu-na) and (3) intranasal priming intramuscular boosting (na-mu). For priming and/or boosting of mice we used 10^6^, 10^7^ or 10^8^ PFU of the vaccine or vehicle as control. In some experiments untreated mice were used as controls. In some experiments consecutive blood samples were collected at 16 hours, 3 days and 10 days post boost. Hamsters were treated with MVA-SARS-CoV-2-S according to mu-na PB protocol. Immunizations were performed using intramuscular applications with vaccine suspension with 10^8^ PFU recombinant MVA-SARS2-S or non-recombinant MVA (mock) into the quadriceps muscle of the left hind leg under isoflurane anesthesia. Boost vaccinations were performed using intranasal applications with vaccine suspension with 10^7^ PFU recombinant MVA-SARS2-S or non-recombinant MVA (mock) under isoflurane anesthesia.

### SARS-CoV-2 Infection

SARS-CoV-2 (BetaCoV/Germany/BavPat1/2020p.1, European Virus Archive Global #026V-03883) received from European Virus Archive was propagated in Vero E6 cells (ATCC #CRL-1586) in Dulbecco’s Modified Eagle’s Medium (DMEM) (Sigma-Aldrich GmbH) supplemented with 2% fetal bovine serum, 1% penicillin-streptomycin and 1% L-glutamine at 37°C. All infection experiments with SARS-CoV-2 were performed in the biosafety level 3 (BSL-3) laboratories at the Research Center for Emerging Infections and Zoonoses (RIZ), University of Veterinary Medicine Hannover, Germany. Hamsters were infected *via* the intranasal route with 1x10^4^ tissue culture infectious dose 50 (TCID50) SARS-CoV-2 under isoflurane anesthesia. After SARS-CoV-2 challenge infection hamsters were monitored daily at least twice for well-being, health constitution and clinical signs, such as body temperature, anorexia, diarrhea/loose stool, vomiting, lethargy/depression and respiratory symptoms using a clinical score sheet. Weights of all hamsters were checked daily.

### Sample Collection From Mice

To label leukocytes present in the blood vessels of lungs, we administered 5 µg of FITC- or BUV717-labeled anti-CD45.2 mAb (clone 30-11) in mice terminally anesthetized with an overdose of ketamine/xylazine. Within 3-5 minutes after antibody administration, blood was collected from saphenous veins and serum was separated by centrifugation and stored at -20°C until further analyses.

After collection of blood, the spleen was resected before broncho-alveolar lavage (BAL) was performed through a plastic catheter clamped into the trachea using three separate lavage steps with 400µl, 300µl and 300µl of PBS each. Collected BAL samples were centrifuged for 10 min at 300 g at 4°C and the supernatant was stored at -20°C for subsequent analysis. BAL cells from pellet were re-suspended in 50 µl of remaining BAL fluid, counted to determine total cell number and immune phenotyped by spectral flow cytometry as described below. Finally, lungs and bronchial lymph nodes were resected. Right lung lobes were dissected and stored in PBS on ice until single cell suspensions were made. Left lung lobes were filled with mixture of PBS : OCT (1:1) and frozen in OCT (Tissue-Tek) on dry ice for histological analysis.

For flow cytometry, single cell suspensions of spleen and bronchial lymph nodes were obtained by meshing the organs through 70 µm cell strainers. Right lung lobes were digested in RPMI supplemented with 10% FCS, 0.025 mg/ml DNAse I (#11284932001, Roche), 0.5 mg/ml Collagenase D (#11088866001 Roche) for 45 minutes at 37°C. Single cells were separated by smashing samples through 70 µm cell strainers. Single cell suspensions of spleen and lung were subjected to hypertonic red blood cell lysis.

In hamster experiments, animals were sacrificed 6 days post SARS-CoV-2 infection and serum as well as lung tissue samples were taken for analysis of virus loads.

### Immunophenotyping Using Spectral Flow Cytometry

For deep phenotyping of immune cells isolated from different organs non-specific antibody binding was blocked by incubating samples 10% rat serum for 10 minutes at 4°C. Next, cells were incubated with MVA- or SARS-CoV-2-S-peptide loaded tetramers for 15 minutes at 37°C. The tetramers were prepared by loading Vaccinia virus WR epitope B8R 20-27 (TSYKFESV) or an 8- amino acid (aa) long immune dominant spike peptide V8L 539-546 (VNFNFNGL) on empty H-2kb tetramers according to the manufacturer’s protocol (Tetramer-Shop). Without washing, antibody mixes were added and cells were incubated for additional 15 minutes at 37°C. The full list of antibodies used is listed in **Supplementary Table S1**. After washing, samples were acquired on Cytek Aurora spectral flow cytometer (Cytek) equipped with five lasers operating on 355nm, 405nm, 488nm, 561nm and 640nm. All flow cytometry data were analyzed using FCS Express V7 (Denovo) or FlowJo V10 (BD).

### Measurement of Viral Burden

Tissue samples of sacrificed hamsters were excised from the right lung lobes and homogenized in 600 µl DMEM containing antibiotics (penicillin and streptomycin, Gibco). Tissue was homogenized using the TissueLyser-II (Qiagen), and aliquots were stored at -80°C. Virus titers were determined on Vero cells as median tissue culture infectious dose (TCID50 units). Briefly, Vero cells were seeded in 96-well plates and serial 10-fold dilutions of homogenized lung samples in DMEM containing 5% FBS. After incubation for 96 hours at 37°C, cytopathic effect in Vero cells was evaluated and calculated as TCID50 unit per gram of hamster lungs by using Reed-Muench method. The detection limit of the assay was 316 TCID50 unit. Thus, for samples where no cytopathic effect was detected, data points were set to 157 TCID50 unit for statical analysis purpose.

### SARS-CoV-2-S Protein Peptides and Epitope Prediction

253 overlapping peptides, each 15 aa long with 10 aa overlap, spanning the whole length of SARS-CoV-2-S or predicted immune dominant peptides were synthesized at >95% purity (GeneScript). For immune dominant peptide prediction, a full-length SARS-CoV-2 sequence was used (YP_009724390.1 from National Center for Biotechnology Information) to predict potential CD8 T cell-immuno dominant 8-11 aa long peptides applying MHC I Binding Tool (with filters summary IC50 < 1000 and Percentile Rank < 10) and MHC I Processing Tool from IEDB (https://www.iedb.org/). For prediction of potential CD4 T cell epitopes, we used IEDB MHC II binding tool (with summary IC50 < 1000) and from that list only 15-aa peptides passing MHC II binding threshold on RankPep (http://imed.med.ucm.es/Tools/rankpep.html) were used. A full list of 13 MHC I and 18 MHC II peptides are shown in **Supplementary Table S2**. All lyophilized peptides were reconstituted at a stock concentration of 50 mg/ml in DMSO (Sigma-Aldrich) except for 9 SARS-CoV-2-S overlapping peptides (number 24, 190, 191, 225, 226, 234, 244, 245 and 246) that were dissolved at 25 mg/ml due to solubility issues. All peptides were stored at -80°C before use.

### T Cell Re-Stimulation Assay

Upon isolation of single-cell suspensions, lung, bronchial lymph node (bLN) and spleen cells were re-suspended in complete RPMI medium [RPMI 1640 (Gibco) supplemented with 10% FBS (GE Healthcare Life Sciences, Logan, UT), 1mM sodium pyruvate, 50 µM β-mercaptoethanol, 1% streptomycin/penicillin (all Gibco)] at concentration of 20 x 10^6^ cells/ml and mixed 1:1 with peptide pools dissolved in complete RPMI containing brefeldin A (Sigma-Aldrich) at final concentrations of 10 µg/ml. In initial experiments, different S- or predicted immunodominant-peptide subpools were tested so that the final concentration of each peptide used for cell stimulation was 2 µg (~1.2 nmol)/ml, except SARS-CoV-2-S overlapping peptides number 24, 190, 191, 225, 226, 234, 244, 245 and 246 that were used at final concentrations of 1 µg/ml due to solubility issues. After initial testing (see **Supplementary Figures 3A–D** and **Supplementary Figures 4A, B**), all other cell stimulations were done with a S-protein overlapping peptides 1-129 (amino acids 1-655) and the all MHC-I and MHC-II predicted immuno-dominant peptides. The maximal total amount of DMSO in the final cell suspension was 5% and the same concentration of DMSO was used as negative control. As positive control, cells were stimulated with Phorbol-12-myristate-13-acetate (PMA; Calbiochem) and ionomycin (Invitrogen) at final concentration of 50 ng/ml and 1500 ng/ml, respectively. After 6 hr incubation at 37°C, 5% CO_2_, cells were collected and stained with antibodies binding to cell surface for 30 minutes at 4°C using anti-CD3ϵ-BV711 (145-2C11,#100349, BioLegend), anti-CD4-PerCP-Cy5.5 (RM4-5, #100540, BioLegend), anti-CD8a-FITC (53-6.7, #100706, BioLegend) or anti-CD8a-APC-R700 (53-6.7,#564983, BD Biosciences) and anti-CD44-BV605 (IM7, #103047, BioLegend) and treated with Zombie NIR™ Fixable Viability Kit (#423106, BioLegend). Afterwards, cells were fixed and permeabilized (eBioscience™ Intracellular Fixation & Permeabilization Buffer Set, Thermo Fisher Scientific) according to the manufacturer’s protocol, before intracellular cytokines were stained using anti-IFNγ-PE (XMG1.2, #505808, BioLegend) and anti-TNFα-APC-Cy7 (MP6-XT22, #560658, BD Biosciences), anti-IL-2-APC (JES6-5H4, #503809, BioLegend), anti-IL-17A-PE-Cy7 (eBio17B7, #25-7177-82, Thermo Fisher Scientific) and anti-IL-5 BV421 (TRFK5, #504311, BioLegend) at room temperature for 30 minutes. After two washes, cells were analyzed by spectral flow cytometry.

### Histology

For histopathology, left hamster lung lobes were fixed by instillation and immersion with 10% buffered formalin. Tissues were subsequently embedded in paraffin and cut into 2 µm thick sections. The lesions were evaluated on hematoxylin and eosin (HE) stained sections in a blinded fashion with a semiquantitative scoring system. Extent of lung inflammation was determined as a median of alveolar, airway and vessel inflammation. Alveolar lesions were evaluated as follows: extent alveolar inflammation and extent alveolar regeneration (0 = no lesions; 1 = minimal, single small foci, max. 1% of tissue section affected; 2 = mild, 2-25%; 3 = moderate, 26-50%; 4 = marked, 51-75%; 5 = subtotal, > 75%. Airway lesions were scored as follows: extent airway inflammation and extent epithelial hyperplasia (0 = none; 1 = minimal, single small foci, max. 1% of tissue section affected; 2 = mild, 2-25%; 3 = moderate, 26-50%; 4 = marked, 51-75% 5 = subtotal, > 75%). Vascular lesions were scored as follows: extent vasculitis (0 = none, 1 = minimal, single vessels, 1% of vessels affected; 2 = mild, 2-25%; 3 = moderate, 26-50%; 4 = marked, 51-75%; 5 = subtotal, > 75%).

### Immunohistology

Frozen tissue blocks of mice lungs were cut in 8µm thick cryo sections and fixed 10 minutes with acetone on ice. Fixed cryo sections containing main stem bronchi were rehydrated for 5 minutes in TBS (PBS, 0.05% Tween) and washed twice with TBS. After washing, cryo sections were blocked (5% rat serum, 5% anti-CD16/CD32 antibody (clone 2.4G2, produced in-house) inTBS) for 15 minutes, incubated for 45 minutes at room temperature with the primary antibodies (**Supplementary Table S3**) and washed twice with TBS. The sections were then stained with DAPI (1 µg/ml) for 3 minutes and washed with TBS again before there were embedded in Moviol. Images were acquired using an Axioscan Z1 (Zeiss). The amount of BALT was quantified as described before (51). In brief, panoramic images of whole sections from different central planes (close to main bronchi and vessels) were collected and analyzed. Individual BALT structures were enumerated, their surface measured, and the cumulative BALT size was calculated as the sum of surface areas of all individual BALT structures present on one central lung section (ZEN 2.3 blue software, Zeiss).

### Immunohistochemistry

Detection of SARS-CoV-2 nucleocapsid protein was performed on formalin-fixed, paraffin-embedded hamster lung tissue using a monoclonal mouse antibody (Sino Biological, 40143-MM0) and the Dako EnVision+ polymer system (Dako Agilent Pathology Solutions) as described (52). Evaluation was performed semiquantitatively for the alveoli and the airway epithelium (0 = no antigen; 1 = minimal, single foci, less than 1% of tissue affected, 2 = mild, 2-25%, 3 = moderate, 26-50%, 4 = severe, 51-75%, 5 = subtotal, >75%). The combined score is the sum of the alveolar and the airway score.

### Plaque Reduction Neutralizing Test for SARS-CoV-2

Sera of infected hamsters were used to analyze neutralization capacity against SARS-CoV-2 as previously described (53). We 2-fold serially diluted heat-inactivated serum samples in Dulbecco modified Eagle medium starting at a dilution of 1:10 in 50 μL. We then added 50 μl of virus suspension (600 TCID50) to each well and incubated at 37°C for 1 h before placing the mixtures on VeroE6 cells. After incubation for 1 h, we washed, cells supplemented with medium, and incubated for 8 h. After incubation, we fixed the cells with 4% formaldehyde/PBS and stained the cells with a polyclonal rabbit antibody against SARS-CoV-2 nucleoprotein (clone 40588-T62, Sino Biological) and a secondary peroxidase-labeled goat anti-rabbit IgG (Dako, Agilent). We developed the signal using a precipitate forming TMB substrate (True Blue, KPL SeraCare) and counted the number of infected cells per well by using the ImmunoSpot^®^ reader (CTL Europe GmbH). The reciprocal of the highest serum dilution allow reduction of >90% plaque formation was calculated as the serum neutralization titer (PRNT90).

### Surrogate Virus Neutralization Test (sVNT)

sVNT test for detection of neutralizing anti-SARS-CoV-2-S antibodies was done as described before (54) with several modifications. Briefly, MaxiSorp 96F plates (Nunc) were coated with recombinant soluble hACE2-Fc(IgG1) protein at 200 ng per well in 100 μl coating buffer (30 mM Na_2_CO_3_, 70 mM NaHCO_3_, pH 9.6) at 4°C overnight. Afterwards, plates were washed with phosphate-buffered saline, 0.05% Tween-20 (PBST) and blocked with 2% bovine serum albumin/2% mouse serum (Invitrogen) (for analysis of sera) or 2% bovine serum albumin (for BAL) in PBS, 0.1% Tween-20 for 1.5 h at 37°C. While plates were blocking, serially diluted and heat-inactivated serum and BAL were incubated with 6 ng recombinant RBD-protein of SARS-CoV-2 Spike S1 carrying a C-terminal His-Tag (Trenzyme) for 1 h at 37 °C. The mixtures were added to hACE2-coated plates and incubated for 1 h at 37°C. His-Tagged RBD-protein without serum or BAL served as control. After extensive washing with PBST, a HRP-conjugated anti-His-tag antibody (clone HIS 3D5, provided by Helmholtz Zentrum München) was added at a final concentration of 1.2 µg/ml and incubated for 1 h at 37°C. Unbound antibody was removed by six washes with PBST. A colorimetric signal was developed on the enzymatic reaction of HRP with the chromogenic substrate 3,3’,5,5’-tetramethylbenzidine (TMB; TMB Substrate Reagent Set, BD Biosciences). An equal volume of 0.2 M H_2_SO_4_ was added to stop the reaction, and absorbance readings at 450 nm and 570 nm were acquired using a SpectraMax iD3 microplate reader (Molecular Devices). Inhibition (%) was calculated as (1 − sample optical density value/negative control optical density value) × 100.

### Enzyme-Linked Immunosorbent Assay (ELISA)

To determine anti-spike IgA and IgG antibodies in sera or BAL samples, ELISA plates were coated overnight at 4°C with 200 ng SARS-CoV-2 trimer in its perfusion conformation (55) fused to mNEONgreen and expressed in Drosophila S2 cells (56) (detailed production will be described elsewhere) in 100µl PBS per well. On the next day, plates were washed three times with washing buffer (PBS, 0.05% (w/v) Tween 20), blocked with 2% (w/v) BSA for 1h at 37°C and incubated for 2h at RT with serial dilutions of sera or BAL. For the detection of anti-spike specific isotype antibodies goat anti-mouse IgG-Fc antibody HRP-conjugated (cat.# 1013-05, SouthernBiotech) and goat anti-mouse IgA-Fc antibody HRP-conjugated (cat.# 1040-05, SouthernBiotech) were used at a final dilution of 1:4000. All assays were conducted in duplicates and quantified using a SpectraMax iD3 microplate reader (Molecular Devices).

### Cytokine Concentration Measurements in Serum

We used Lunaris multiplex assays (Ayoxxa) for analysis of serum cytokine levels at indicated time points in the short prime-boost protocol treated mice. The 7-plex custom assay included IFN-γ, IL-1β, TNF-α, IL-6, IL-12p70, IL-17 and IL-2 and the 4-plex assay included IL-4, IL-5, IL-10 and IL-13. For each assay, serum samples were diluted 1:2 with assay buffer and 5 µl of diluted sample was incubated overnight on the chip. Assay was performed according to manufacturer’s instruction and analysed on Lunaris Reader 384 (Ayoxxa). The data evaluation with automated data adjustment was done using Lunaris Analsysis Suite v1.4 (Ayoxxa). Limits of the detection were 0.52 pg/ml (IFNγ); 7.23 pg/ml (IL-1β); 3.74 pg/ml (IL-2); 3.58 pg/ml (IL-6); 7.16 pg/ml (IL-12p70); 4.99 pg/ml (IL-17A); 1.54 pg/ml (TNF-α); 101.25 pg/ml (IL-4); 3.30 pg/ml (IL-5); 9.03 pg/ml (IL-10); and 12.40 pg/ml (IL-13). For visualization, data for each cytokine were expressed as fold increase to the mean value of the buffer treated mice at 10 days post boost.

### Statistics

Statistical analysis was done using Prism 7 or 8 (GraphPad). Statistical analysis was done on log-transformed values using ordinary or Welch’s ANOVA followed by Dunnett’s T3 multiple comparisons test. P values < 0.05 were considered statistically significant.

## Results

### Intranasal MVA-SARS-2-S Immunization Induces BALT Formation

**Figure 1:**
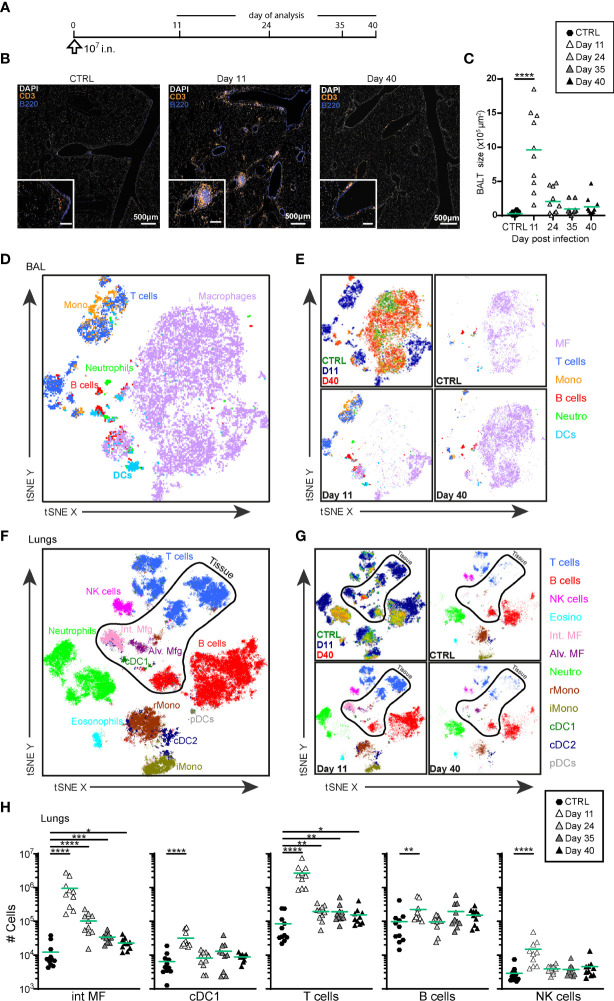
Intranasal priming with MVA-SARS-2-S induces BALT and transient recruitment of T cells and macrophages in the lung. **(A)** Immunization protocol scheme. **(B)** Representative photomicrographs of lung sections reveal induction of BALT peaking at d11 after vaccine application. **(C)** Quantification of cumulative BALT size per lung section averaged on 3-4 lung sections per mouse. **(D–G)** Cellular composition of broncho-alveolar lavage (BAL; **(D, E)** and lung **(F, G)** analyzed by spectral flow cytometry and depicted as tSNE plot. Cluster identities were revealed by manual gating as shown in **Supplementary Figures 1A, B**; antibodies used are listed in **Supplementary Table S1** (panel 1). Cells inside the area delineated by the black line were negative for anti-CD45-FITC antibodies i.v injected 3 -5 min before mice were sacrificed and are thus considered to be from the lung parenchyma. Cells outside that area were CD45-FITC^+^ and considered to be within or in close vicinity to blood vessels. **(D, F)** Representative tSNE plot of concatenated samples from one mouse each sacrificed before (control) or 11 or 40 days after vaccine application; colors refer to indicated cell populations; antibodies used are listed in **Supplementary Table S1** (panel 2). **(E, G)** Upper left, concatenated data as in **(D, F)**, colors indicate different mice analyzed. Upper right and bottom plots: de-concatenated data, colors indicate cell populations. **(H)** Absolute cell numbers of interstitial macrophages (int MF), type 1 conventional dendritic cells (cDC1), NK, T and B cells in lung. **(C, H)** Pooled data from 3-4 experiments with n = 10 per group. **(C, H)** Individual values (symbols) and mean group value (lines). Statistical analysis was done on log-transformed values using ordinary or Welch’s ANOVA followed by Dunnett’s T3 multiple comparisons test. *p < 0.05, **p < 0.01, ***p < 0.001, ****p < 0.0001.

In agreement with our previous results (44), i.n. administration of MVA-SARS-2-S (**Figure 1A**) led to BALT formation at day 11 p.i., although some B and T cells remained scattered diffusely around vessels and bronchioles (**Figures 1B, C** **)**. Over time, BALT contracted, and very late BALT structures at day 40 p.i. were mainly composed of T cells (**Figure 1B**).

Global high-dimensional mapping of broncho-alveolar lavage fluid (BAL) cells and digested lung tissue with two different panels by spectral flow cytometry revealed profound changes in immune cell composition induced by vaccination (**Figures 1D–G**; **Supplementary Figures 1A, B** and **Supplementary Table S1**). At day 11 post i.n. vaccination we observed marked accumulation T cells, B cells and monocytes in BAL (**Figures 1D, E** and **Supplementary Figure 1C**). These cell populations decreased in absolute numbers over time and by day 40 p.i. and, as in the non-immunized control mice, macrophages became again the dominating cell population (**Figures 1D, E** **and** **Supplementary Figure 1C**). Similarly, at d11 p.i. we observed massive recruitment of T cells, B cells, macrophages, monocytes, NK cells, and dendritic cells in lung tissue, as revealed by the absence of staining of an anti-CD45 mAb that had been intravenously (i.v.) injected 10min before the mice were sacrificed; **Figures 1F–H** and **Supplementary Figure 1D**). The number of T cells and interstitial macrophages within the tissue remained elevated even 40 days post i.n. MVA-SARS-2-S delivery (**Figure 1H** and **Supplementary Figure 1D**), reflecting the presence of BALT observed by histology (**Figure 1B**). Collectively, these data demonstrate that single i.n. administration of MVA-SARS-2-S induces an accumulation of T and B cells and the formation of BALT in lungs.

### Single i.n. Application of MVA-SARS-2-S Induces Strong Cellular but Weak Humoral Spike-Specific Immune Responses

The increased numbers of T cells at d11 p.i. was primarily due to the accumulation of CD4^+^ as well as CD8^+^ effector/effector memory (CD62L^-^CD44^hi^) cells (**Supplementary Figure 2** and **Supplementary Table S1**). Their presence in the lungs as well as in bronchial lymph nodes (bLNs) and spleen (**Supplementary Figure 2**) demonstrated that a single i.n. MVA-SARS-2-S immunization induces not only local but also systemic immune responses.

**Figure 2:**
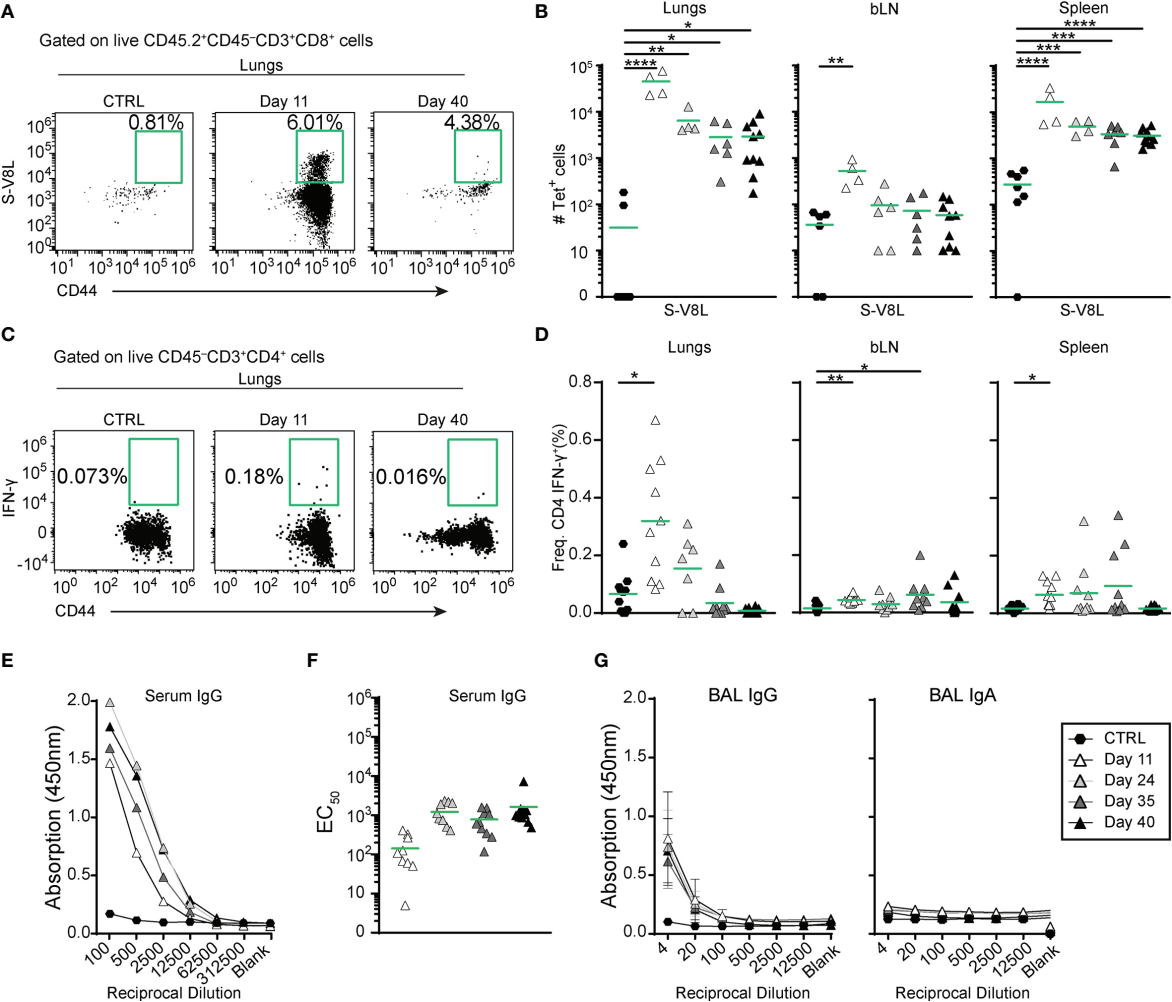
A single intranasal vaccine application induces local and systemic spike-specific cellular and humoral responses. **(A, B)** Spike-specific CD8^+^ T cells accumulate in in the organs at different time points after vaccine application. **(A)** Representative dot plots and percentage of tetramer^+^ lung CD8^+^CD44^hi^ T cells in groups indicated. **(B)** Absolute cell counts of spike-specific CD8^+^CD44^hi^ T cells in different organs. **(C, D)** Spike-specific CD4^+^ T cells transiently accumulate in lungs and spleens up to day 35 after immunization. **(C)** Representative dot plots and percentages of IFN-γ-expressing lung CD8^+^CD44^hi^ T cells of groups indicated after *ex vivo* re-stimulation with the pool of spike-specific and immnodominant peptides (**Supplementary Table S2**) for 6hr. **(D)** Frequencies of IFN-γ-expressing CD4^+^CD44^hi^ cells in different organs isolated from mice at indicated time points after i.n. vaccine administration. **(E–G)** Spike-specific antibodies in serum **(E, F)** and broncho-alveolar lavage fluid (BAL; **(G)** measured by ELISA. **(E)** Mean OD group values of serial serum dilutions and **(F)** and individual EC_50_ values for Spike-specific serum IgG for the groups indicated. **(G)** Mean group OD values in serial BAL dilutions for Spike-specific IgG (left) and IgA (right). **(B, D, F)** Pooled data from 3-4 experiments with n = 4-10 mice per group. Individual values (symbols) and mean group value (line). Statistical analysis was done on log-transformed values using ordinary or Welch’s ANOVA followed by Dunnett’s T3 multiple comparisons test. *p < 0.05, **p < 0.01, ***p < 0.001, ****p < 0.0001.

To specifically measure S-specific CD8^+^ T cell responses, we established an *ex vivo* stimulation assay with 10-amino acid overlapping 15-mer peptides covering the entire S-protein. Quantification of intracellular interferon γ (IFN-γ) expression by flow cytometry revealed that immunization with MVA-SARS-2-S induced CD8^+^ T cells specific to peptides 1-129 (amino acids 1-655), covering almost the entire S1 domain of the S protein (**Supplementary Figures 3A, B**). In addition, the Immune Epitope Database (IEDB) (57) allowed us to identify an immunodominant SARS-CoV-2-S H2-Kb epitope at S539-546 (VNFNFNGL; S-V8L) of the S1 C-terminal domain (**Supplementary Figures 3C–F** and **Supplementary Table S2**). Integrating S-V8L tetramers in spectral FACS analysis, we found that S-specific CD8^+^ T cells peaked in lungs, BALs, bLNs and spleens at d11 p.i. and slowly decreased to day 40 (**Figures 2A, B** and **Supplementary Figure 3G**). Importantly, even at this time-point their number remained significantly elevated in lungs, BAL, and spleen compared to non-immunized controls, indicating development of immunological memory. The majority of those cells produced tumor necrosis alpha (TNF-α) in addition to IFN-γ upon re-stimulation with S protein-derived peptides (**Supplementary Figure 3H**). Of note, the kinetics of S-specific CD8^+^ cells matched the kinetics of CD8^+^ T cell specific for a known immuno-dominant epitope of MVA in C57BL/6 mice (TSYKFESV; MVA-B8R) (58) (**Supplementary Figures 3I, K**).

Analysis of *ex vivo* cultured cells stimulated with S protein-derived peptides further revealed that S-specific IFN-γ producing CD4^+^ T cells were also present in lungs, bLNs, and spleens of immunized animals up to day 24 post i.n. vaccination (**Figures 2C, D** and **Supplementary Figure 4A, B**). Importantly, we could not detect IL-5 producing CD4^+^ T cells upon *ex vivo* re-stimulation (**Supplementary Figure 4C**). Together these data indicate that i.n. MVA-SARS-2-S vaccination induces, in addition to CD8 immunity, also specific T helper type 1 (Th1), but not Th2 responses.

Profiling of B cell responses after a single i.n. dose of the vaccine revealed increased numbers of germinal center (GC) B cells (CD19^+^CD138^-^IgD^-^IgM^-^GL7^+^CD73^+^) in bLN and lungs but not in spleen (**Supplementary Figures 4D, E**). Despite the increase in GC B cell counts, anti-S antibodies were only present at relatively low levels in all mice (**Figure 2E**). Mean EC_50_ values were in the range of 1:100 (10^2^) at day 11 and increased approximately 10 fold at later time points analyzed (**Figure 2F**). Similarly, BAL from immunized mice also possessed low anti-S IgG antibody titers that stayed constant over time, but no anti-S IgA antibodies could be detected (**Figure 2G**). Together, these data indicate that a single i.n. immunization with MVA-SARS-2-S induces strong local and systemic cellular but only moderate humoral immune responses directed against the spike protein.

### Intranasal Boosting With MVA-SARS-2-S Induces Strong Cellular and Humoral Immune Responses in Lungs Irrespective of the Priming-Route

**Figure 3:**
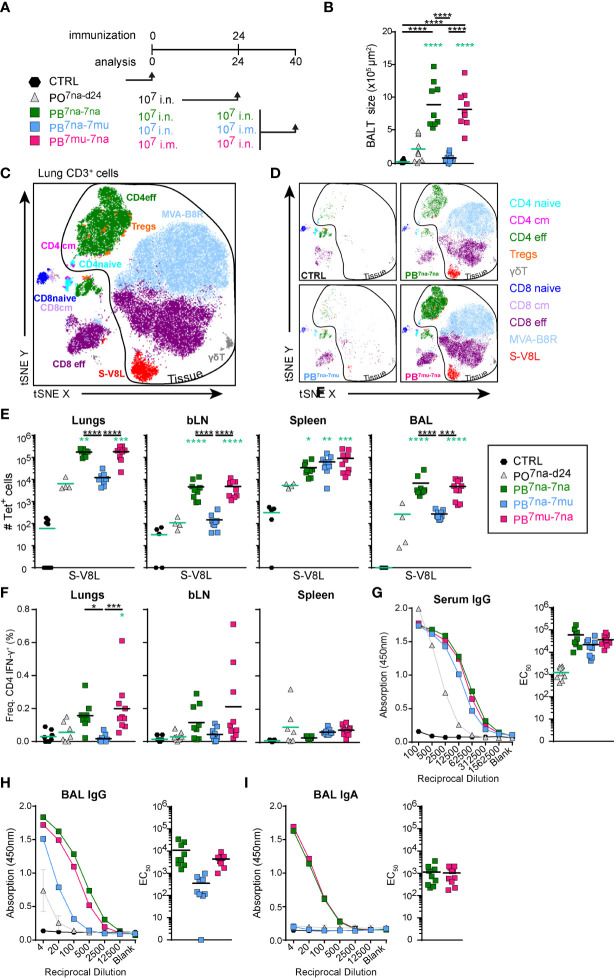
Intranasal boost with MVA-SARS-2-S induces strong local and systemic cellular immune responses irrespectively of the route of priming. **(A)** Immunization protocol scheme. **(B)** Cumulative size of BALT structures averaged on 3-4 central lung sections per mouse. **(C, D)** Composition of lung CD3^+^ T cells analyzed by spectral flow cytometry and depicted as tSNE plot. Cluster identities were revealed by manual gating as shown in **Supplementary Figure 2**; antibodies used are listed in **Supplementary Table S1** (panel 3). Cells inside the area delineated by the black line were negative for anti-CD45-FITC antibodies i.v. injected 3 -5 min. before mice were sacrificed and are thus considered to be from the lung parenchyma. Cells outside that area were CD45-FITC^+^ and considered to be within or in close vicinity to blood vessels. **(C)** tSNE plot of concatenated FCS files from one representative mouse of each prime-boost group immunized with 10^7^ PFU vaccine and one non-immunized control mouse. **(D)** Individual tSNE plots of de-concatenated FCS files from **(D)**. **(E)** Absolute counts of CD8^+^ T cells specific for S-V8L evaluated by tetramer staining in tissues and groups indicated. **(F)** Frequencies of IFN-γ^+^ CD4 T cells in organs indicated determined by intracellular cytokine staining after re-stimulation with the pool of S_1-129_ together with immnodominant peptides and brefeldin A (**Supplementary Table S2**) for 6hr. **(G)** Spike-specific antibodies in serum and **(H, I)** BAL determined by ELISA. Mean OD group values of serial serum and BAL dilutions and individual EC_50_ values for Spike-specific IgG in serum **(G)** and BAL **(H)** and Spike-specific IgA in BAL **(I)** are shown for the groups indicated analyzed at day 40. Pooled data from 3-4 experiments with n = 10 per group. (B,E–I) Individual values (signs) and mean group value (line). Statistical analysis was done on log-transformed values using ordinary or Welch’s ANOVA followed by Dunnett’s T3 multiple comparisons test. Green stars - difference to the mice immunized with PO^7na-d24^; Black stars - difference between different treatment groups as indicated with line. *p < 0.05, **p < 0.01, ***p < 0.001, ****p < 0.0001. **(B, E–I)** Data from control mice and mice immunized with 10^7^ IU MVA-SARS-2-S are identical to that shown in **Figure 1C**; **(B, D–F)**.

Since MVA vector vaccines are notoriously known to induce strong immune responses in boost vaccination protocols (59–61), we boosted mice at day 24 and analyzed them at day 40 (**Figure 3A**). According to our central hypothesis that delivery of the vaccine to the respiratory tract is required for the induction of protective immunity in the lung, we compared single intranasal application using 10^7^ PFU (PO^7na-d24^) with three different prime-boost immunization protocols (1): priming and boosting by i.n. vaccination (PB^7na-7na^) (2), i.n. priming followed by an intramuscular boost (PB^7na-7mu^), and (3) i.m. priming followed by an i.n. boost (PB^7mu-7na^).

Of note, when compared to PO^7na-d24^ group, increased amounts of BALT were only induced by i.n. but not intramuscular boost, independent of the priming route applied (**Figure 3B**). This finding was confirmed by flow cytometry quantifying the major immune cell subsets in BAL and lung (**Supplementary Figures 5A, B** **)**. The composition of T cells residing in the lung parenchyma was comparable between the different prime-boost immunization protocols but groups that received the i.n. boost had considerably more cells than primed-only mice or mice in PB^7na-7mu^ group (**Figures 3C, D** **)**. Likewise, CD8^+^ T cells specific for S-V8L as well as MVA-B8R were strongly increased in lungs, bLNs and BALs of the PB^7na-7na^ and PB^7mu-7na^ groups, while such differences were not found in spleens (**Figure 3E** and **Supplementary Figure 5C**). Further, *in vitro*-re-stimulation with S-specific peptides identified increased frequencies of IFNγ-producing CD4^+^ T cells in the lungs (**Figure 3F**).

In addition to stronger cellular immune responses, all prime-boost protocols induced also significantly higher S-specific IgG titers in serum compared to the mice receiving only a single i.n. vaccination (**Figure 3G**). Similar results were found for anti-S IgG in the BAL, although mice receiving intramuscular boost developed clearly lower titers compared to intranasal boosted mice (**Figure 3H**). Most importantly, protocols applying i.n. boost (sPB^7na-na^ and PB^7mu-na^) induced anti-S IgA antibodies in the BAL (**Figure 3I**), the antibody isotype that protects respiratory mucosal surfaces from infection with air-borne pathogens.

### High Prime Dose Induces Tissue Specific S-Specific T-Cells

**Figure 4:**
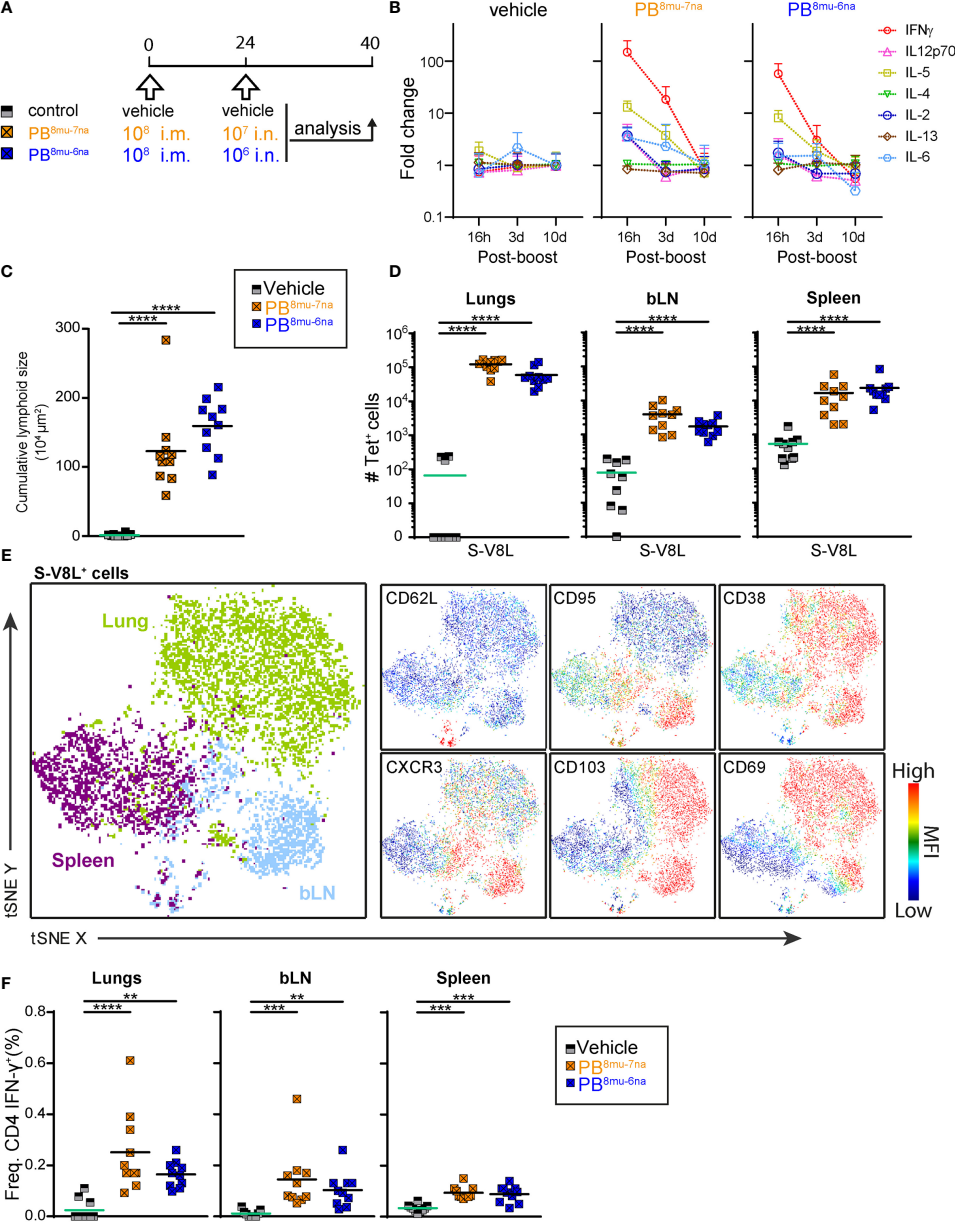
Intramuscular-intranasal prime-boost immunization protocol generates S-specific T cells with tissue resident memory phenotype. **(A)** Immunization protocol scheme. **(B)** Relative change of serum cytokine concentrations at times indicated after i.n. boost determined by Ayoxxa’s LUNARIS™ multiplex biomarker platform; cytokine concentrations are given **Supplementary Table S4**. **(C)** Cumulative size of BALT structures averaged from 3-4 central lung sections per mouse. **(D)** Absolute counts of S-V8L-T^+^CD44^hi^ CD8 T cells in different organs of vaccination groups indicated. **(E)** tSNE plot illustrating CD3^+^CD8^+^CD44^hi^ S-V8L-tet^+^ T cells from various organs. Shown are concatenated FCS file from each organ indicated of 2 representative mice immunized by the sPB^8mu-7na^ protocol and analyzed by spectral flow cytometry. Colors refer to cells originating from indicated tissues (left, big plot) or to expression levels with red indicating high and blue low expression (6 smaller plots). **(F)** Frequencies of CD44^hi^IFN-γ^+^ CD4 T cells analyzed by intracellular cytokine staining after stimulation with the pool of S_1-129_ together with immnodominant peptides and brefeldin A (**Supplementary Table S2**) for 6hr. Pooled data from 2 experiments with n = 6-10 per group. Individual values (signs) and mean group value (line). Statistical analysis was done on log-transformed values using ordinary or Welch’s ANOVA followed by Dunnett’s T3 multiple comparisons test. **p < 0.01, ***p < 0.001, ****p < 0.0001.

Our results so far revealed that prime-boost protocols give stronger immune responses than prime-only vaccine delivery and that intranasal boosting induced the best local adaptive immune response. Next, we investigated immune responses after priming with optimal i.m. priming dose (10^8^ PFU) for MVA-based vaccines (50, 62), followed by lower booster doses of i.n. vaccine delivery (10^7^ or 10^6^ PFU) (**Figure 4A**). To monitor cytokines induced by i.n. vaccine delivery, we assessed serum concentrations of 11 cytokines at different times point after boosting (**Supplementary Table S4**). The most prominent changes were observed for IFN-γ that was elevated dose-dependently but transiently, increasing approximately 150-fold and 60-fold with 10^7^ PFU and 10^6^ PFU, respectively at 16 hr post boost and returning to the levels of control mice by day 10 post boost (**Figure 4B** and **Supplementary Table S4**). Similar patterns, albeit to a much lower extent were also observed for the T cell proliferation cytokine IL-2, the Th1-cytokine, IL-12p70, as well as IL-6 (approximately a 3-fold increase with the higher vaccine dose). Interestingly, i.n. vaccine boost also induced transient and dose-dependent increase in serum levels of the Th2 cytokine IL-5 (13-fold and 8-fold with higher and lower vaccine dose, respectively; **Figure 4B**), a cytokine important for maturation of IgA-secreting B cells (63). Importantly, other Th2 cytokines such as IL-4 and IL-13 were not affected (**Supplementary Table S4**). In line with no effects on body weight and body temperature, the concentrations of the classical pro-inflammatory cytokines TNF-α and IL-1β, the regulatory cytokine IL-10 or Th17 cytokine IL-17A were not affected by the boost (**Supplementary Figures 6A, B** and **Supplementary Table S4**) underlining the excellent tolerability of MVA-SARS-2-S after i.n. boost application.

Interestingly, the higher booster dose (10^7^ PFU) induced slightly less BALT than the lower dose (10^6^ PFU; **Figure 4C**), accompanied by strong accumulation of all major immune cell subsets in BAL and lung in both boosted groups (**Supplementary Figures 6C–E**). In contrast, the higher booster dose induced slightly more S-V8L CD8^+^ T cells in lung and bLN, while both protocols were equally efficient in the spleen (**Figure 4D**). Likewise, both vaccine doses induced an identical TNF-α/IFN-γ production profile in CD8^+^ T cells following *ex vivo* peptide re-stimulation (**Supplementary Figure 6F**). Detailed flow cytometric profiling of S-V8L CD8^+^ T cells indicated that they cluster according to the organ of origin (**Figure 4E**). Importantly, expression of Trm markers CD103 and CD69 was largely restricted to lung and bLN CD8^+^ T cells (**Figure 4E** and **Supplementary Figure 6G**).

Similarly to CD8^+^ T cell responses, the low and the high booster doses were equally efficient in inducing S-specific Th1 cells in lung, bLN and spleen (**Figure 4F**). Of note, we found no evidence of IL-5-secreting Th2 or IL-17A-secreting Th17 cells in any organ upon re-stimulation with spike-derived peptides (**Supplementary Figures 6H, I** **)**. In summary, both booster protocols, PB^8mu-7na^ and PB^8mu-6na^, induced very similar and effective cellular immunity.

### MVA-SARS-2-S Boosting Induces High Titers of Neutralizing Anti-SARS-CoV-2-S Antibodies

**Figure 5:**
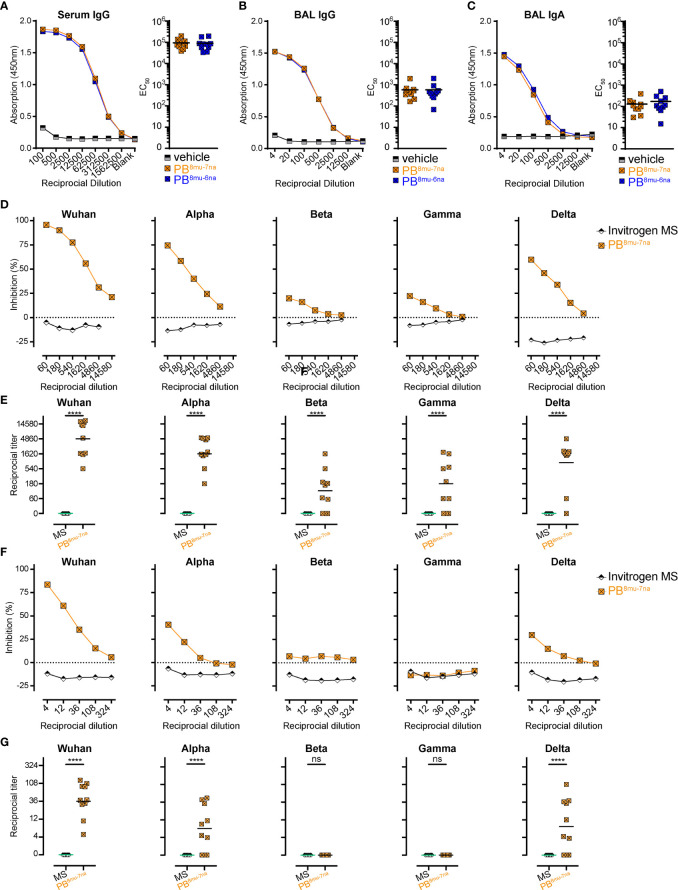
Intranasal boosting with MVA-SARS-2-S induces high titer of neutralizing antibodies in lung and serum. **(A–C)** Spike-specific antibodies in serum **(A)** and BAL **(B, C)** measured by ELISA. Mean group OD values for S-specific IgG **(A, B)** and IgA **(C)** determined on serially diluted serum **(A)** and BAL **(B, C)** of indicated groups 14 days post boost (d40). **(D–G)** Neutralizing antibodies in serum **(D, E)** and BAL **(F, G)** measured by surrogate virus neutralization test (sVNT). **(D, F)** Inhibition of binding interaction of SARS-CoV-2 spike-RBD from different SARS-CoV-2 variants of concern (VoC) with ACE2 by addition of sera **(D)** or BAL **(F)** of immunized or control mice. Assay performed in triplicate; mean percentages of neutralization. **(E, G)** Reciprocal titers of neutralizing antibodies against SARS-CoV-2 VoCs from serum **(E)** or BAL **(G)** determined as the dilution retaining binding reduction > mean+2SD of non-immunized control mice. **(A–G)** Pooled data from 2-4 experiments with n = 10 per group. **(E, G)** Individual values (signs) and mean group value (line). Statistical analysis was done using Chi-square test for trend, ns, not significant, ****p < 0.001.

In addition to S-specific T cells, both booster doses were equally efficient in inducing anti-S antibodies in serum and BAL (**Figures 5A–C**). We further measured neutralization capacity of those antibodies in samples of the PB^8mu-7na^ group using our recently established surrogate virus neutralization tests (sVNT) for different SARS-CoV-2 VoC (14, 54, 64). The sVNT is based on ELISA technology and allows high-throughput quantitative analysis of neutralizing antibody (nAb) levels by measuring the reduction in the binding of the receptor-binding-domain (RBD) of SARS-CoV-2-S protein to ACE2 *in vitro*. Compared to serum samples from unimmunized mice, all mice from PB^8mu-7na^ group developed high amounts of nAb in the serum (**Figures 5D, E** **)**. Furthermore, these antibodies also efficiently blocked alpha and delta variants, while the protection against beta and gamma variants was less effective but still present in 7 out of 10 immunized mice (**Figures 5D, E** **)**. Importantly, nAb against Wuhan, alpha, and delta but not against beta and gamma variants were detectable also in BAL (**Figures 5F, G** **)**. Together these results underline the power of inhalative vaccine delivery for the induction of systemic and mucosal protective humoral responses.

### I.m. Priming Followed by an i.n. Boost With MVA-SARS-CoV-2-S Efficiently Protects Golden Syrian Hamsters From SARS-2-S Infection

**Figure 6:**
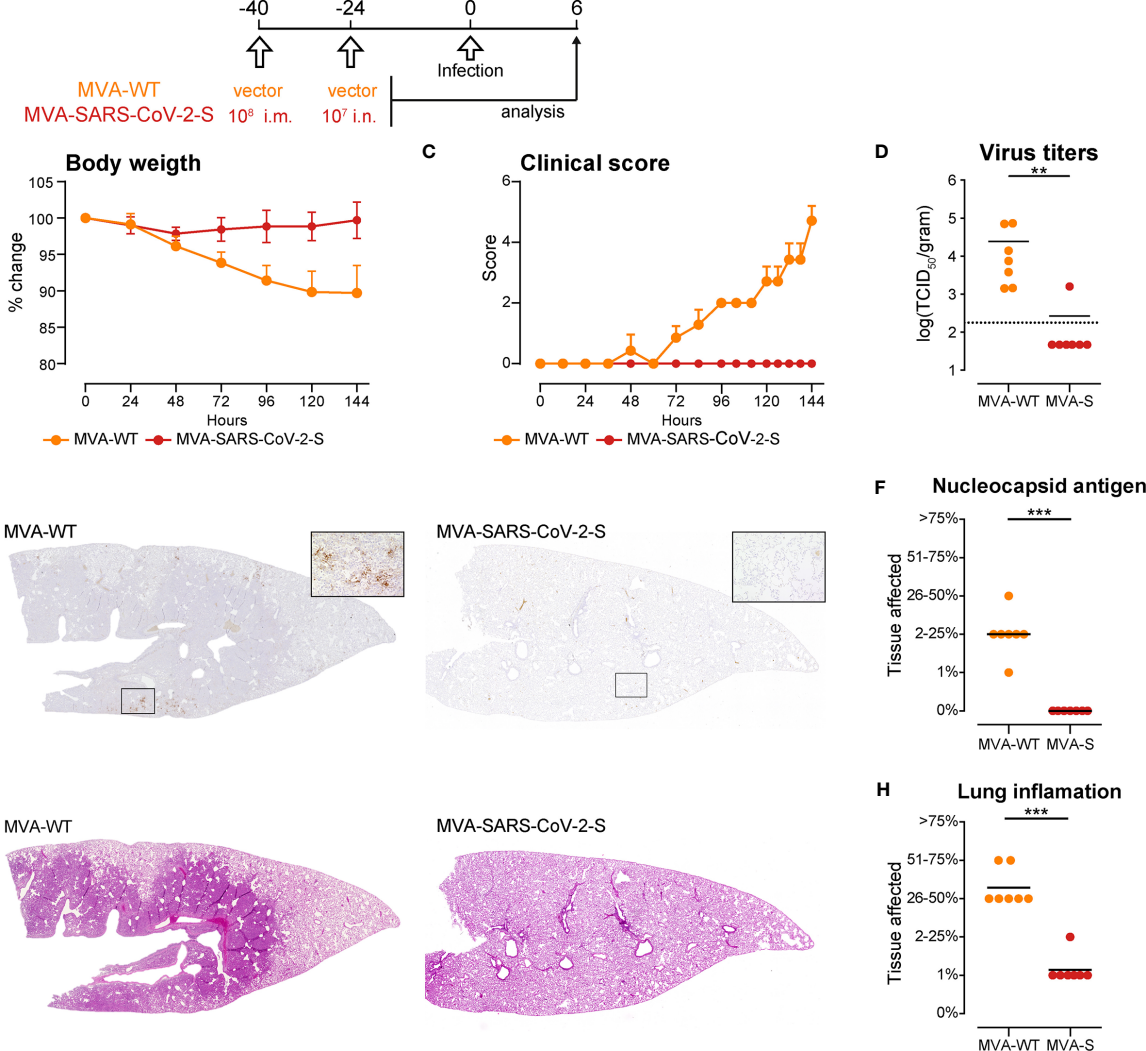
Intranasal immunization with MVA-SARS-2-S protects hamsters from SARS-CoV-2 infection. **(A)** Immunization and infection protocol scheme. **(B, C)** Relative body weight **(B)** and clinical scores **(C)** of hamsters after infection with SARS-CoV-2. **(D)** SARS-CoV-2 virus titers in the lungs measured by quantitative RT-PCR. **(E)** Representative stitched photomicrographs showing an overview of the entire left lung lobe stained for SARS-CoV-2 nuceloprotein (brown staining). Original magnification 40x, inset 400x. **(F)** Semiquantitative scoring of SARS-CoV-2 nuceloprotein per lung section averaged on 2 lung sections per hamster. **(G)** Representative stitched photomicrographs showing an overview of the entire left lung lobe stained with hematoxylin and eosin **(H, E)** indicate severe inflammation in the lungs of hamsters immunized with control, MVA-WT virus, which is almost not existent in the lungs of hamsters immunized with MVA-SARS-CoV-2. Original magnification 40x. **(H)** Lung inflammation scores calculated as described supplemental methods. **(B–H)** Pooled data from one experiment with n = 7 per group. Individual values (signs) and mean group value (line). Statistical analysis was done using Mann-Whitney t test. **p < 0.01, ***p < 0.001.

SARS-CoV-2 cannot bind to mouse ACE2 rendering mice resistant to lethal infection. Thus, we tested the protective capacity of the i.m.-i.n. vaccination regime with MVA-SARS-CoV-2-S in a hamster model of SARS-CoV-2 infection (65, 66). We immunized two groups of golden Syrian hamsters, one with MVA-SARS-CoV-2-S and the other with MVA vector (MVA-WT), using the PB^8mu-7na^ protocol, which proved to be the most effective protocol for in mice. Forty days post prime, the hamsters were infected with 1 × 10^4^ tissue culture infectious dose 50 (TCID50) SARS-CoV-2 (isolate BavPat1/2020 isolate, European Virus Archive Global #026V- 03883). The course of infection was tracked for 6 days by animal weight loss as well as changes in spontaneous behavior and general condition summarized in a clinical score (**Figure 6A**). In contrast to MVA-WT-immunized control hamsters, vaccination with MVA-SARS-2-S completely blocked development of clinical signs of infection (**Figures 6B, C** **)**. Moreover, at day 6 post SARS-CoV-2 infection, when substantial virus RNA loads were found in the lungs of MVA-WT-immunized control hamsters, minimal SARS-CoV-2 titers were found in only one of 7 lungs from MVA-SARS-2-S–immunized animals (**Figure 6D**). Similarly, using immunohistochemistry we detected SARS-CoV-2 nucleoprotein in the lungs of all MVA-WT-immunized control hamsters but in none of the MVA-SARS-2-S–immunized animals (**Figures 6E, F** **)**. These spots of viral replication in the lungs of all MVA-WT-immunized control hamsters were usually located at areas of prominent leukocyte infiltration and consolidation that affected more than one quarter of the lungs (**Figures 6G, H** and **Supplementary Figure 7**). In contrast, 6 out of 7 hamsters immunized with MVA-SARS-2-S had only single small foci of mild inflammatory infiltrates, affecting max. 1% of the lungs (**Figures 6G, H** and **Supplementary Figure 7**). These data indicated that immunity induced by respiratory vaccination with MVA- SARS-2-S so rapidly cleared SARS-CoV-2 infection that infection and inflammation could not spread to large lung areas. Interestingly, we did not found any evidences of BALT structures in any of the analyzed groups. While it is possible that the SARS-CoV-2 infection led to the dissolution of the BALT structures, these data could also be a consequence of species-dependent differences in the MVA-induced BALT development that require further investigation.

**Figure 7:**
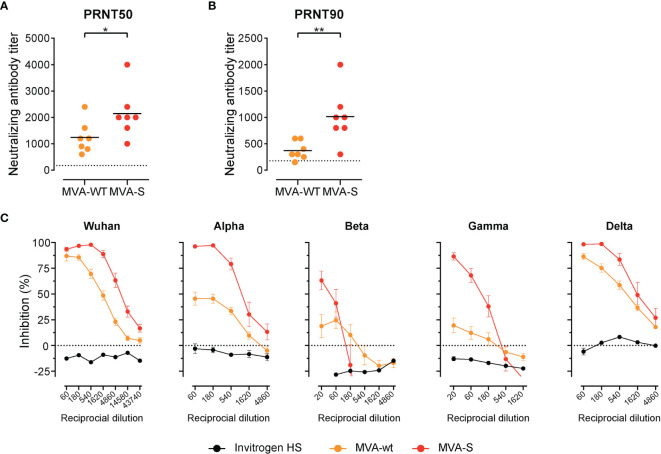
High titer of neutralizing antibodies in serum of immunized hamsters. **(A, B)** Levels of neutralizing Spike-specific antibodies in serum of hamsters immunized with MVA-WT or MVA-SARS-CoV-2 and measured using SARS-CoV-2 plaque reduction neutralization test (PRNT). Reciprocal titer values that result in 50% **(A)** or 90% **(B)** reduction in virus infectivity determined on serially diluted serum of indicated groups 20 days post boost (d46) and 6 days post SARS-CoV-2 infection. **(C)** Neutralizing antibodies against different SARS-CoV-2-S variants measured by surrogate virus neutralization test (sVNT). Inhibition of binding interaction of SARS-CoV-2 spike-RBD with ACE2 by addition of sera of non-immunized and uninfected hamsters (Invitrogen HS), MVA-WT, and MVA-SARS-CoV-2-S immunized and SARS-CoV-2 infected hamsters. Assay performed in duplicate for each sample; mean percentages of neutralization per group ± SD are shown. **(A–C)** Pooled data from 2-4 experiments with n = 7 per group. Individual values (signs) and mean group value (line). Statistical analysis was done using Mann-Whitney t test. *p < 0.05, **p < 0.01.

Lastly, we measured levels of neutralizing antibodies in sera of control and MVA-SARS-2-S–immunized hamsters. SARS-CoV-2 S protein-pseudotyped-vesicular stomatitis virus vector-based neutralization assay revealed that at this time point post infection the MVA-WT immunized animals also had detectable nAb against SARS-CoV-2 in serum (**Figures 7A, B** **)**, which is in line with previous reports (67, 68). Not surprisingly, the nAb levels in MVA-SARS-CoV-2-S immunized animals were significantly higher (**Figures 7A, B** **)**. In line with the data from vaccinated mice, sVNT revealed that these nAbs are very effective against Wuhan, alpha, and delta SARS-CoV-2 variants, while protection against beta and gamma variants was less prominent but still clearly observable in MVA-SARS-CoV-2-S vaccinated hamsters (**Figure 7C**).

## Discussion

Our study showed that respiratory delivery of a MVA-SARS-2-S vaccine efficiently protected hamsters from SARS-CoV-2 infection. Importantly, not only was the virus completely cleared from the lungs but vaccinated hamsters also had only minimal traces of resolving lung inflammation. The rapid clearance of SARS-CoV-2 combined with the minimal immune response can only be attributed to the existence of two major pillars of adaptive immunity in the respiratory tract: the local production and secretion of IgA antibodies and tissue resident memory T cells directed against the spike protein. Indeed, our extensive profiling in mice indicated that an intra nasal vaccine boost is crucial for the induction of high titers of neutralizing anti-S-RBD antibodies in BAL. Importantly, these antibodies were successful in neutralizing two predominant SARS-CoV-2 VoC, alpha and delta. These neutralizing anti-S-RBD antibodies play a crucial role in preventing (re-)infections with the virus, as they block binding of the S protein to its cellular receptor ACE2, thus preventing virus cell entry (54, 64, 69, 70). Moreover, i.n. boosting led to a massive accumulation of spike-specific CD8^+^ T cells expressing CD103 and CD69, markers for tissue residency in the lungs (71). In case neutralizing antibody protection is breached, these CD8^+^ T cells can rapidly be re-activated for instant and efficient virus control (72). Although tissue-resident memory CD8 T cells might have limited longevity in the lung (73, 74), they appear to be crucial for limiting the severity of lung pathology induced by immune response to viral infection (75). Vaccination with MVA-SARS-2-S *via* the respiratory tract, therefore, provided the local armamentarium needed for effective protection against SARS-CoV-2, which is in agreement with previous reports indicating that i.n. administration of MVA-based vaccines can protect against various respiratory pathogens (38, 43, 76–78).

Additionally, intranasal delivery of MVA-SARS-2-S in mice also led to the induction of BALT, as reported earlier for MVA, the backbone for the current vaccine (44–46). Interestingly, BALT induced by i.n. booster delivery of MVA-SARS-2-S affected larger lung areas and persisted longer than BALT induced by a single i.n. dose of the vaccine, which is in line with the hypothesis that the extent and the nature of the inflammatory stimuli control BALT formation (79–81). On the other hand, hamsters immunized with the same vaccine did not develop BALT, suggesting species-specific mechanisms important for BALT induction after MVA administration. Of note, lack of BALT did not affect vaccine efficacy. BALT does not seem to contribute to the maintenance of IgA-producing plasma cells (30, 79) or tissue resident memory CD8^+^ T cells (82). However, BALT serves an important site for T cell activation as well as B cell selection and maturation fighting pathogens that reach the lower respiratory tract (44, 79). MVA-SARS-2-S-induced BALT might therefore provide the infrastructure that also facilitates immune responses to other pathogens. In line with this idea, the presence of BALT has been shown to significantly accelerate clearance of different respiratory viruses, including influenza, SARS-CoV-1 and pneumovirus (83).

Interestingly, intramuscular boosting of intranasally primed mice failed to induce neutralizing antibodies and to stimulate the expansion of spike-specific resident T cells within lungs, further emphasizing the requirement of an antigen-driven local re-activation of virus-specific lymphocytes for the establishment of a protective environment in the lung. In contrast, the route used for priming seems to be of less importance, as we only observed rather subtle differences in the adaptive immune response between PB^na-na^ and PB^mu-na^ protocols (**Figure 3**). These results confirm the findings that local vaccination induces anti-virus immunity at the organism level (84) and suggest that local (intranasal) boosting helps to target the immune system to the particular organ.

Profound immune responses in the lung induced by intranasal boosting were not achieved at the expense of systemic immunity. Induction of systemic immunity is important because infections with SARS-CoV-2 are not restricted to the respiratory system but can affect basically all other organs. Serum titers of S-specific IgG antibodies induced by the various PB protocols of the present study are comparable to titers reported after intramuscular or intraperitoneal prime-boost immunization with other MVA-based SARS-CoV-2 vaccines (50, 60, 61). Our data clearly indicate that upon successful systemic priming even low vaccine doses are sufficient for efficient boosting. Furthermore, we used the sVNT to quantify anti-S nAbs, which is done in a species-independent manner and thus allows comparison of immune responses between different species. It is therefore of interest to note that mice i.n. boosted with 10^7^ PFU by the various PB protocols developed nAb titers approximately 10-fold higher than those present in convalescent individuals recovering from severe COVID-19 (54) and in the range of those vaccinees that received heterologous ChAdOx1-nCov-19 (Vaxzevria, ChAd)/BNT162b2 (Comirnaty, BNT) vaccination (14). These data strongly indicate that humoral immune responses induced by MVA-SARS-2-S could efficiently protect against SARS-CoV-2 infection.

The MVA-SARS-2-S vaccine used in this study was very well tolerated, similarly to other MVA vector vaccines delivered *via* the respiratory route in preclinical (42, 43) and clinical (48) studies. Importantly, our data show that once the immune system has been successfully primed even low doses (10^6^ PFU) of the MVA vector vaccines are sufficient to allow for full boosting *via* the respiratory tract. Of note, systemic priming followed by aerosol boosting of a MVA vector vaccine for tuberculosis, MVA85A, led to transient but clinically significant respiratory adverse effects in some volunteers (49). These adverse effects can probably be attributed to the high vaccine dose (5x10^7^ PFU) used for aerosol delivery and/or to cellular impurities possibly present in the vaccine. Although MVA-based vaccines appear to be very well tolerated after intramuscular application (48, 49, 85), the respiratory route of vaccine delivery is not well established in humans. Therefore, it will be important to carefully assess safety of the respiratory delivery of MVA-SARS-2-S in clinics and to monitor for any potential adverse effects, such as Bell’s palsy reported after intranasal application of inactivated flu vaccine (86).

In summary, we demonstrate efficacy of MVA-SARS-2-S after intranasal delivery that is mediated by fulminant induction of cytotoxic CD8^+^ T cells, a strong Th1 response and high titers of neutralizing antibodies against SARS-CoV-2 in serum and BAL. Together with the results of other preclinical studies (23–27), these data support respiratory vaccine delivery as a promising application route to interfere with the spread of SARS-CoV-2 and to prevent COVID-19.

## Data Availability Statement

The original contributions presented in the study are included in the article/**Supplementary Material**. Further inquiries can be directed to the corresponding authors.

## Ethics Statement

The animal studies were reviewed and approved by Niedersächsisches Landesamt für Verbraucherschutz und Lebensmittelsicherheit (LAVES), Lower Saxony, Germany.

## Author Contributions

RF conceptualized work. RF and BB designed study and wrote manuscript. GSu, AV, AT, JS, GK, and LL prepared and characterized the MVA-SARS-2-S vaccine. TK and GSs constructed, produced and purified SARS-CoV-2-mNEON protein. BB, IO, JB-M, IS, SH, MP, GP, HG, RG-J, SW, AB, JR, and CR performed experiments. BB, IO, JB-M, IS, SH, MP, GP, HG, and RG-J analyzed data and drafted figures. JBM designed figure layout with help from IO, BB, and RF. MC and WB performed histopathology and immunohistochemistry for virus antigen of hamster lung sections. TT, CMzN, D-LS, SC, and AV performed immunization and challenge of hamster, collected samples, determined viral load in hamster lungs and/or performed PRNT with hamster sera. All authors contributed to manuscript draft correction. All authors contributed to the article and approved the submitted version.

## Funding

This work was supported by Deutsche Forschungsgemeinschaft, (DFG, German research Foundation) Excellence Strategy EXC 2155 “RESIST” to RF and TK (Project ID39087428); by funds of the state of Lower Saxony (14-76103-184 CORONA-11/20) to RF and TK; by funds of the BMBF ("NaFoUniMedCovid19” FKZ: 01KX2021; Projects “COVIM”and “B-FAST”) to RF and TK and Deutsche Forschungsgemeinschaft, Projektnummer 158989968 - SFB 900 (Projects B1 to RF and B10 to TK); Projektnumber Fo 334/7-1 and the Bundesministerium für Bildung und Forschung (DZIF 01.92100 and 01KX2026 to GSu, ZOOVAC 01KI1718 and RAPID 01KI1723G to AV) and by a BMBF (Federal Ministry of Education and Research) project entitled RAPID (Risk assessment in re-pandemic respiratory infectious diseases), 01KI1723G and by the Ministry of Science and Culture of Lower Saxony in Germany (14 - 76103-184 CORONA-15/20) to WB.

## Conflict of Interest

The authors declare that the research was conducted in the absence of any commercial or financial relationships that could be construed as a potential conflict of interest.

## Publisher’s Note

All claims expressed in this article are solely those of the authors and do not necessarily represent those of their affiliated organizations, or those of the publisher, the editors and the reviewers. Any product that may be evaluated in this article, or claim that may be made by its manufacturer, is not guaranteed or endorsed by the publisher.
