# Supplementary material for: Intranasal Delivery of MVA Vector Vaccine Induces Effective Pulmonary Immunity Against SARS-CoV-2 in Rodents
Source: Front Immunol. 2021 Nov 11;12:772240. doi: 10.3389/fimmu.2021.772240 (PMC8632543; doi:10.3389/fimmu.2021.772240)
Supplement: Supplementary file 1 [file DataSheet_1.docx]

Supplementary Figures and Tables


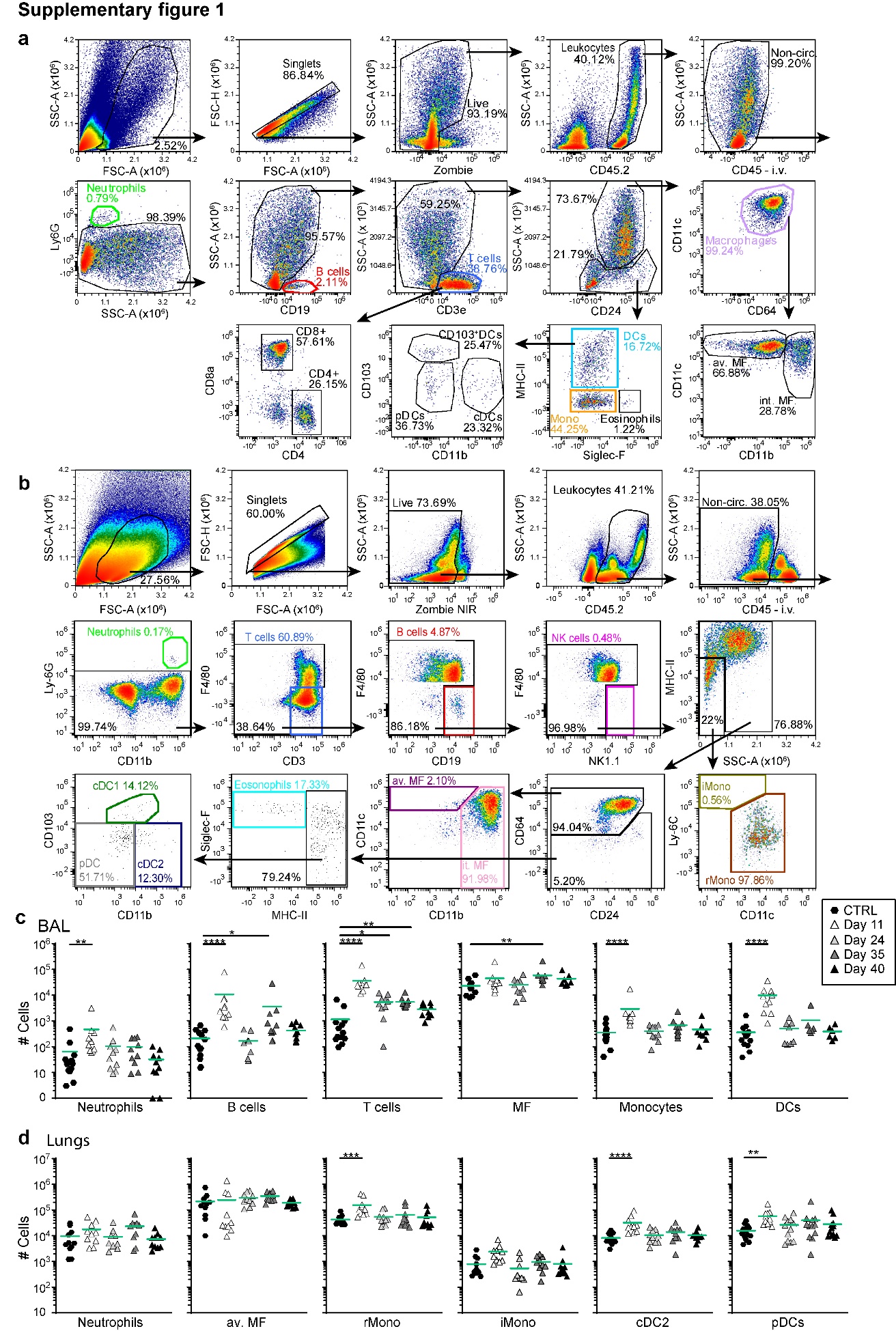


**Supplementary Figure 1. Gating strategy and quantification of myeloid cells recruitment to the lung induced by single intra nasally (i.n.) deliverd dose of MVA-SARS-CoV-2-S.**

Mice were immunized i.n. at day 0 with 10^7^ PFU and analyzed at day 11, 24, 35 and 40 post-immunization. (Immunization protocol scheme in Fig. 1a). **a, b)** Pseudocolor plots showing gating strategy for indicated cell populations in (**a**) broncho-alveolar lavage fluid (BAL) analyzed with 19 panel-1 mAb (Supplementary Table S1), and (**b**) lungs analyzed with 19 panel-2-mAb (Supplementary Table S1). Data shown are from a representative mouse at day 11 after vaccine application. **c, d**) Absolute cell counts of cell populations indicated present in (**c**) BAL and (**d**) lungs of mice analyzed at time points indicated after a single i.n. application of 10^7^ PFU of the vaccine. Pooled data from 3-4 experiments with n = 10 per group. Shown are individual values (symbols) and mean group values (line). Statistical analysis was done on log-transformed values using ordinary or Welch’s ANOVA followed by Dunnett’s T3 multiple comparisons test. * p < 0.05, ** p < 0.01, *** p < 0.001, **** p < 0.0001. Abbreviations: Macrophages (MF), dendritic cells (DCs), alveolar macrophages (av. MF), resident Monocytes (rMono), induced Monocytes (iMono), type 2 conventional dendritic cells (cDC2s) and plasmacytoid dendritic cells (pDCs).

**
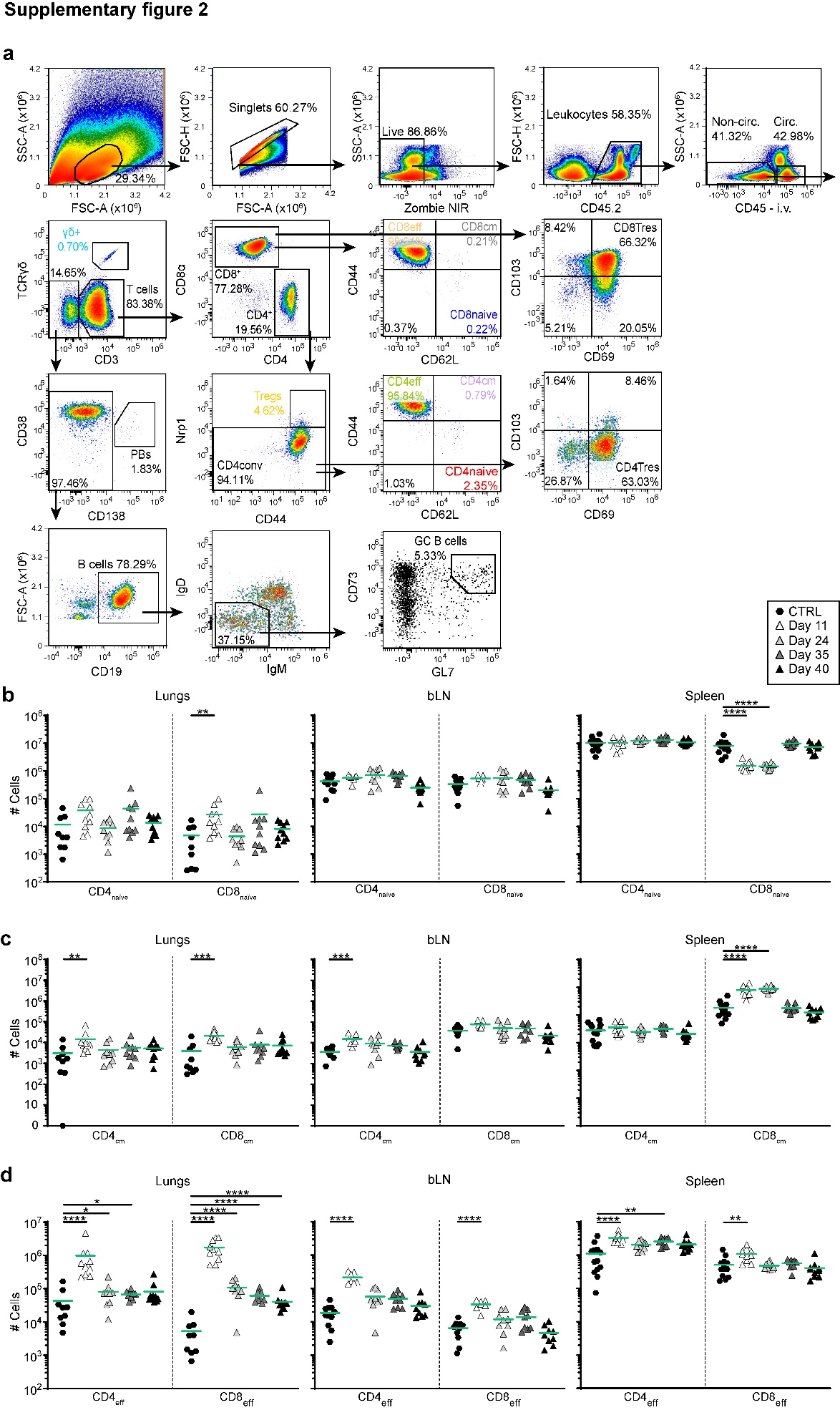
**

**Supplementary Figure 2. T cells transiently accumulate in the lung after a single intra nasally (i.n.) delivered dose of MVA-SARS-CoV-2-S.**

**a)** Gating strategy for indicated cell populations in lung stained with 27 panel-3 mAb (Supplementary Table S1). Pseudocolor plots show representative data from one mouse analyzed at day 11 post vaccine delivery. **b-d)** Absolute cell counts of (**b**) naïve (CD62L^+^CD44^low^), (**c**) central memory (T_CM_; CD62L^+^CD44^hi^), and () effector / effector memory (CD62L^-^CD44^hi^)CD4^+^ and CD8^+^ T cell populations gated as depicted in (**a**). Organs analyzed and time points of analysis as (days post immunization) as indicated. Pooled data from 3-4 experiments with n = 10 per group. Shown are individual values (symbols) and mean group values (lines). Statistical analysis was done on log-transformed values using ordinary or Welch’s ANOVA followed by Dunnett’s T3 multiple comparisons test. * p < 0.05, ** p < 0.01, *** p < 0.001, **** p < 0.0001.


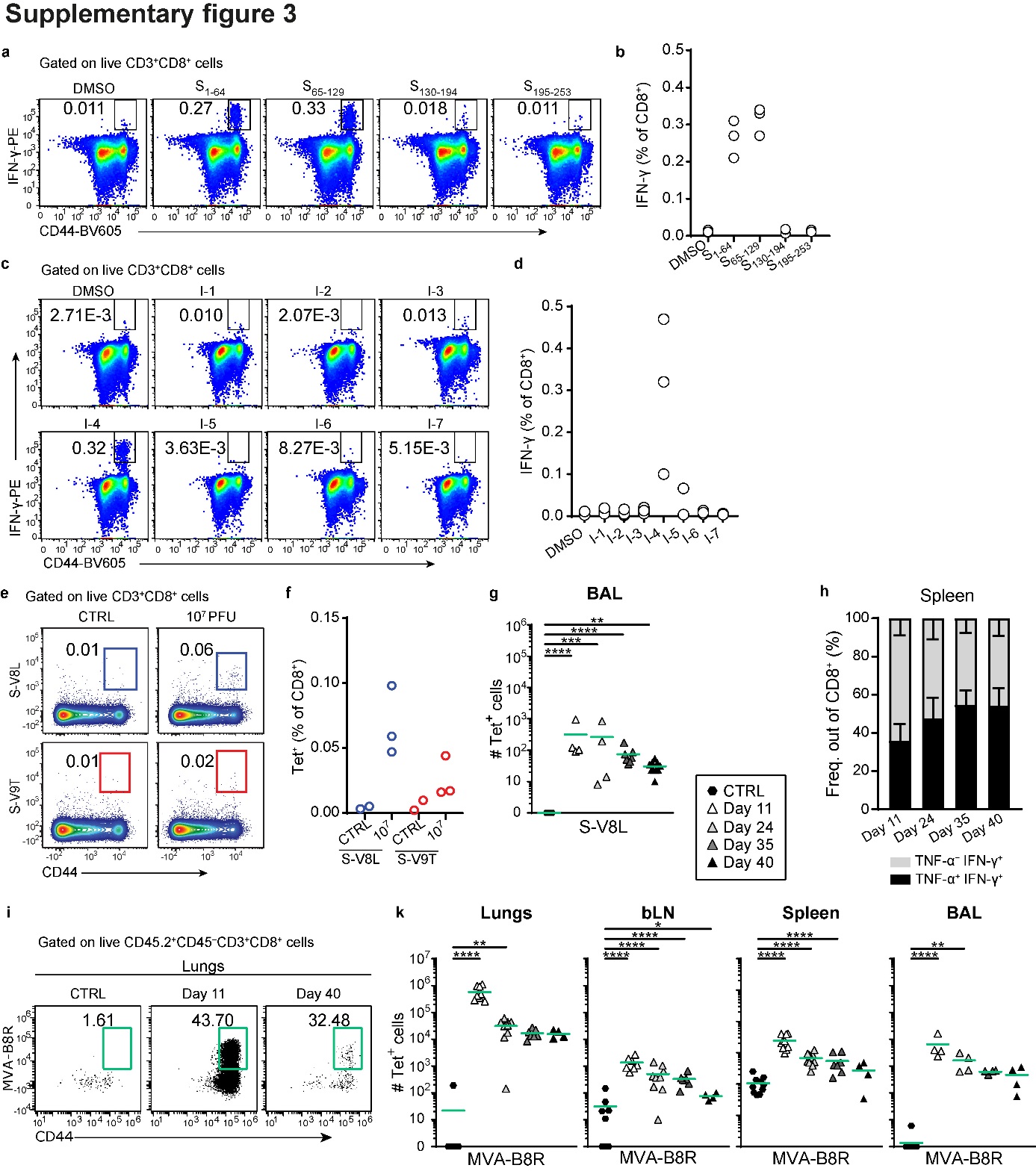


**Supplementary Figure 3. A single intra nasally (i.n.) delivered dose of MVA-SARS-CoV-2-S induces local and systemic MVA-SARS-CoV-2-S-specific T cell responses.**

**a-b**) Intracellular IFN-γ expression in splenocytes stimulated with *ex vivo* with DMSO or different peptide pools of SARS-CoV-2-Spike protein for 6 hr. Underscript numbers correspond to the order of consecutive peptides in the total S- pool consisting of 15-amino-acid-long peptides with 10 amino acid overlap. (**a**) Representative pseudocolor plots and (**b**) summary graph showing frequencies of IFN-γ^+^ cells of spleen CD8^+^CD44^hi^ T cells isolated from spleen. One representative experiment with mice at 11 days post vaccination is shown. Dots represent mice; representative from one of two independent experiments. **c-d)** Immuno-dominant peptide I-1 to I-7, composed of a single peptide or sequence variations of individual peptides as described in Table S2. (**c**) Representative pseudocolor plots and (**d**) summary graph showing frequencies of CD8^+^CD44^hi^IFN-γ^+^ T cells isolated from the spleen at day 24 after i.n. vaccine application and stimulated with peptides indicated. Dots represent mice; data from one of two independent experiments. **e,f)** Flow cytometric analysis of CD8^+^ splenocytes stained with tetramers loaded with the two peptides - S-V8L or S-V9T from I4 (Supplement Table S2). (**e**) Representative pseudocolor dot plots and (**f**) frequencies of tetramer^+^ CD8^+^ splenocytes from non-treated mice or mice i.n. immunized by vaccine 24 days earlier; dots represent mice; representative from two independent experiments. **g)** Absolute cell counts of S-V8L-tetramer^+^ CD8^+^ T cells in BAL at time points indicated following after intranasal immunization. Pooled data from 2-4 experiments with n = 4-10 per group; individual values (symbols) and mean group values (lines) are shown. **h)** Frequency of TNF-α expressing cells from CD8^+^CD44^+^IFN-γ^+^ T cells. For cytokine stimulation, splenocytes were *ex vivo* stimulated with the pool of S_1-129_ together with immnodominant peptides (Table S2) for 6hr. Data are presented as mean frequency ± SD. **i,k**) Analysis of CD8^+^ T cells specific for the MVA vector (TSYKFESV; B8R) in organs and time points after i.n. vaccine delivery. (**k**) Representative dot plots and (**l**) absolute cells counts of B8R-tetramer^+^ cells. Pooled data from 2-4 experiments with n = 4-10 per group. Shown are individual values (symbols) and mean group values (lines) are shown. (**g,I,k**) Statistical analysis was done on log-transformed values using ordinary or Welch’s ANOVA followed by Dunnett’s T3 multiple comparisons test. * p < 0.05, ** p < 0.01, *** p < 0.001, **** p < 0.0001.

**
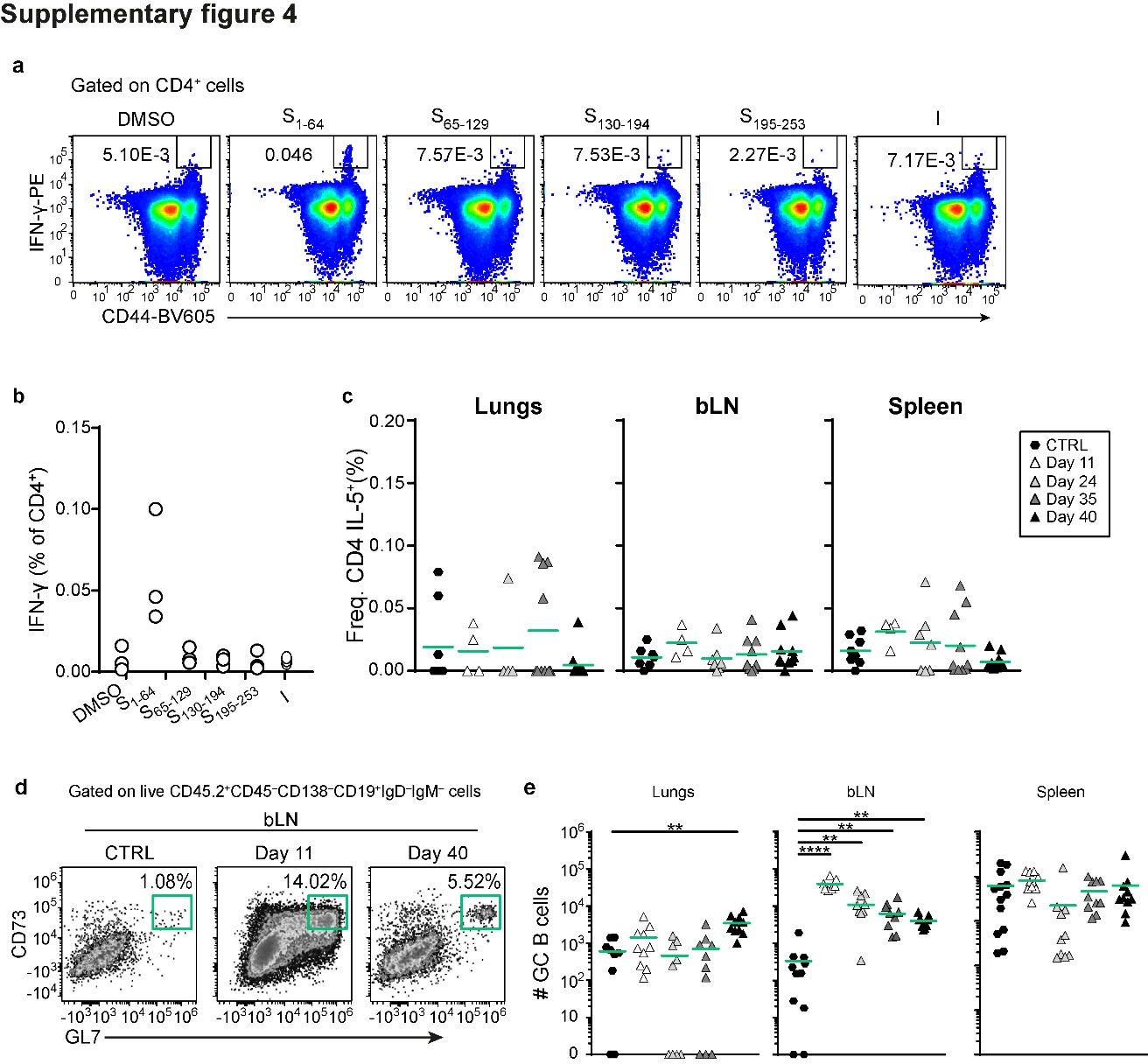
**

**Supplementary Figure 4. Single i.n. application of MVA-SARS-CoV-2-S induces type 1 but not type 2 spike-specific CD4+ T helper cell responses.**

**a-b**) Analysis of intracellular IFN-γ expression in CD4^+^ splenocytes re-stimulated *ex vivo* with DMSO or indicated peptide pools of SARS-CoV-2-Spike protein for 6 hr. S-protein peptide subpools as described for Supplementary Figure 3a, immunodominant peptide pool [I] contains all peptides listed in Table S2. (**a**) Representative pseudocolor plots and (**b**) summary graph showing frequencies of IFN-γ^+^ cells of spleen CD4^+^CD44^hi^ T cells isolated from one representative mouse at day 11 post i.n. immunization and re-stimulation indicated; dots represent mice; representative of two independent experiments. **c)** Frequency of IL-5^+^ CD4^+^ T cells in different organs isolated from mice at indicated time points after i.n. immunization. Cells were *ex vivo* stimulated with the pool of S_1-129_ together with immunodominant peptides (Supplementary Table S2) for 6hr. **d)** Representative dot plots of germinal center B cells (CD73^+^GL7^+^) pre-gated on CD19^+^CD138^-^IgD^-^IgM^-^ cells from bronchial lymph node (bLN) analyzed at indicated time points after a single intranasal application 10^7^ PFU of MVA-SARS-2-S and from a non-immunized control mouse. **e)** Absolute cell counts of germinal center B cells in different organs analyzed at time points indicated. Data are from 2-4 experiments with n=4-10 per group; symbols represent mice, lines group means. Statistical analysis was done on log-transformed values using ordinary or Welch’s ANOVA followed by Dunnett’s T3 multiple comparisons test. ** p < 0.01, **** p < 0.0001.


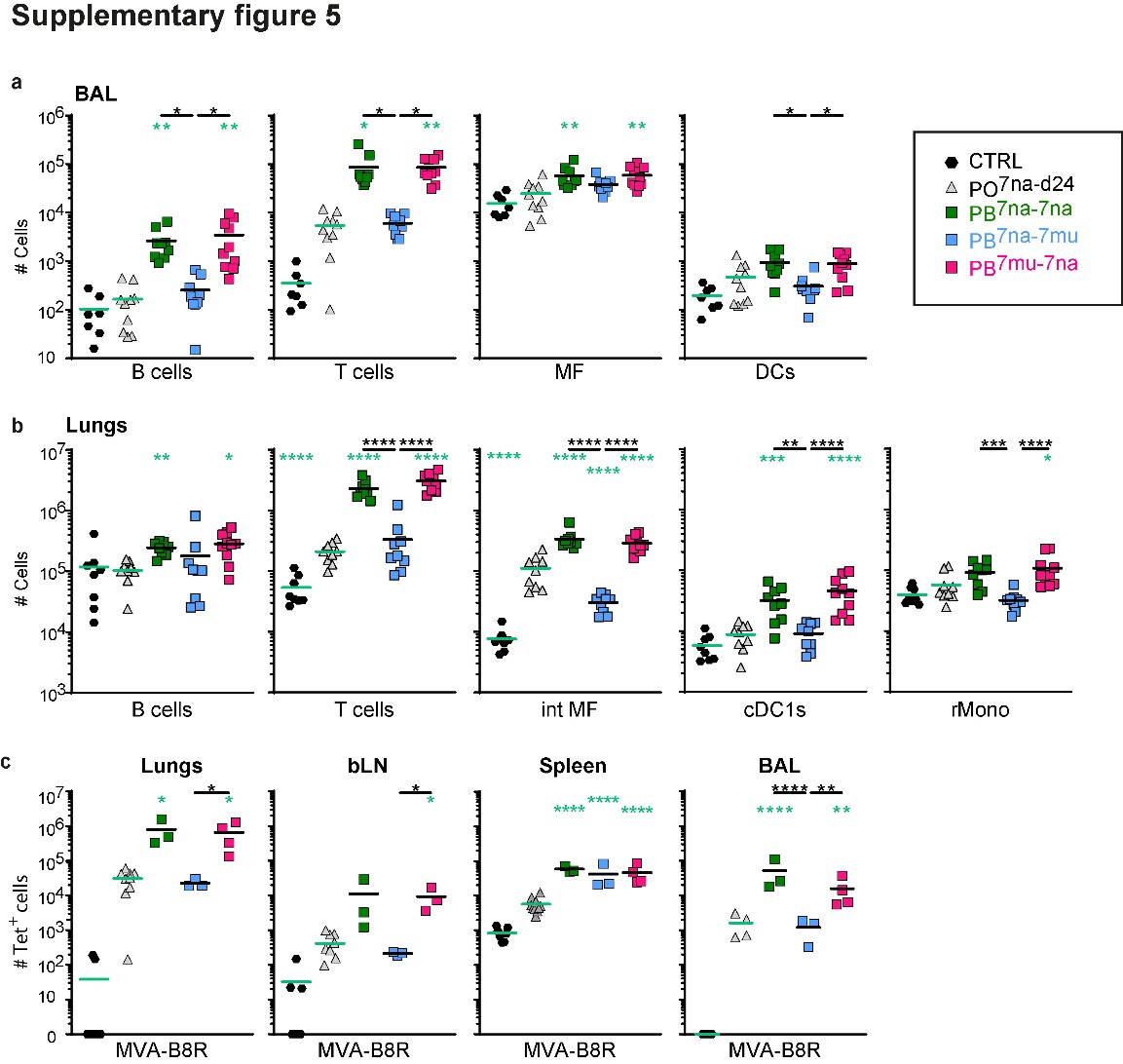


**Supplementary Figure 5. Intranasal but not intramuscular boost with MVA-SARS-2-S induces strong local and systemic cellular immune responses irrespectively of the route of priming.**

Mice were immunized i.m. (pink symbols) or i.n. (green and blue symbols) at day 0 with 10^7^ PFU. Primed mice were boosted at day 24 with 10^7^ PFU i.m. (blue symbols) or i.n. (green and pink symbols) and analyzed at day 40 (Immunization protocol scheme in Fig. 3a). **a,b)** Absolute cell counts of indicated immune cell sub populations in broncho-alveolar lavage fluid (BAL; **a**) and lung (**b**). **c)** Absolute cell counts of MVA vector-specific (MVA-B8R) CD8 T cells analyzed by tetramer staining in organs indicated. Pooled data from 3-4 experiments with n = 10 per group; symbols represent mice, lines group means. Statistical analysis was done on log-transformed values using ordinary or Welch’s ANOVA followed by Dunnett’s T3 multiple comparisons test. Green stars - difference to the mice immunized with PO^7na-d24^; Black stars - difference between different treatment groups as indicated with line. * p < 0.05, ** p < 0.01, *** p < 0.001, **** p < 0.0001. **a-c)** Data from control mice and mice immunized with 10^7^ IU MVA-SARS-2-S are identical to that shown in Sup.Fig. 1c (a); Fig. 1c (b); and Sup.Fig. 3k (c). Abbreviations as in Supplementary Fig. 1 and Fig. 1.

**
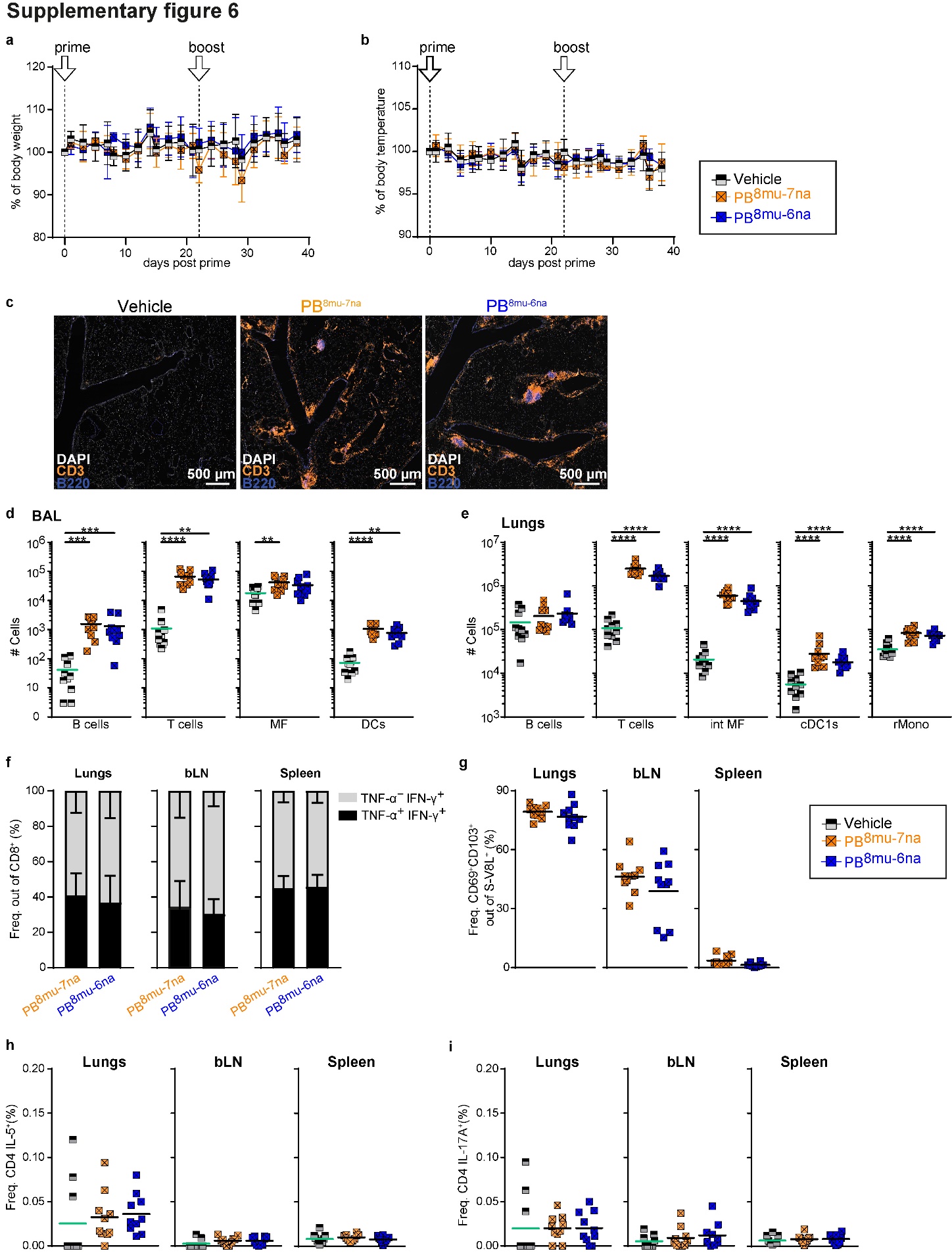
**

**Supplementary Figure 6. No adverse side effects following intra muscular priming with 10^8^ and subsequent intra nasal boosting with 10^7^ or 10^6^ PFU of MVA-SARS-CoV-2-S**

Mice were immunized i.m. at day 0 with 10^8^ PFU (orange and blue symbols) or vehicle only (black/gray symbol). Primed mice were boosted at day 24 with 10^7^ PFU (orange symbols), 10^6^ PFU (blue symbols) or vehicle only (black/gray symbols) and analyzed at day 40 (Immunization protocol scheme as shown in Fig. 4a). **a,b)** Relative body weight (**a**) and body temperature (**b**) over the course of experiment. **c)** Representative photomicrographs of lung sections reveal BALT in mice immunized with MVA-SARS-CoV-2-S. **d,e)** Absolute cell counts of major immune cells subsets in BAL (**d**) and lung (**e**). **f)** Relative distribution of CD8^+^ T cells responding to *ex vivo* re-stimulation with the pool of S_1-129_ together with immnodominant peptides (Supplementary Table S2) for 6h with regard to TNF-α and IFN−γ production. **g)** Frequency of CD8^+^ T cells specific for S-V8L showing a tissue resident phenotype (CD103+CD69+). **h,i)** Frequency of IL-5- (**h**) and IL-17-producing (**i**) CD4 T cells from the organs indicated following *ex vivo* re-stimulation with the S_1-129_ peptide pool together with immnodominant peptides (Supplementary Table S2) for 6hr. Pooled data from 2 experiments with n = 10 per group. Individual values (symbols) and mean group values (lines) shown. Statistical analysis was done on log-transformed values using ordinary or Welch’s ANOVA followed by Dunnett’s T3 multiple comparisons test. ** p < 0.01, *** p < 0.001, **** p < 0.0001. Abbreviations as in Supplementary Fig. 1 and Fig. 1.


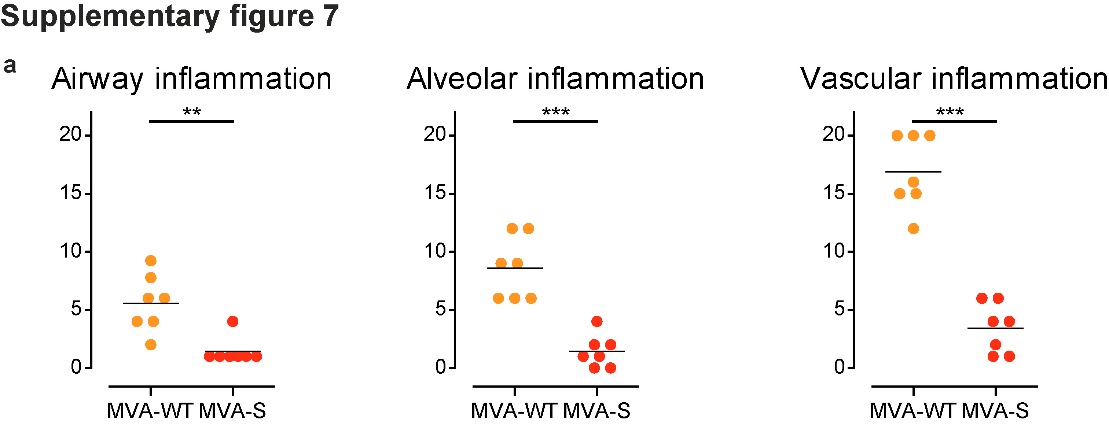


**Supplementary Figure 7. Individual scores of airway, alveoli, and vascular inflammation determined as described in supplemental methods.** Pooled data from 1 experiment with n = 7 per group. Individual values (signs) and mean group value (line). Statistical analysis was done using Mann-Whitney t test. ** p < 0.01, *** p < 0.001.

**Supplementary Table S1.** List of reagents used for flow cytometry. Panel 1 antibodies were used for bronchial alveolar lavage (BAL), panel 2 for lung innate leucocyte population and panel 3 for lung lymphocyte population staining. N/A – not applicable

| Antigen | Clone | Fluorophore | Catalogue No. | Company | Panel |
| --- | --- | --- | --- | --- | --- |
| B220 | RA3-6B2 | BUV496 | 612950 | BD | 2 |
| CCR4 | 2G12 | PerCP-Cy5.5 | 131220 | Biolegend | 3 |
| CD103 | 2E7 | BV 421 | 121422 | Biolegend | 2 |
| CD103 | 2E7 | BV 711 | 121435 | Biolegend | 3 |
| CD103 | 2E7 | BV 785 | 121439 | Biolegend | 1 |
| CD11b | M1/70.15 | PE-Texas Red | RM2817 | Invitrogen | 1,2 |
| CD11c | N418 | BV 650 | 117339 | Biolegend | 1,2 |
| CD127 | SB/199 | PE-Cy5 | 121124 | Biolegend | 3 |
| CD138 | 281-2 | BV 785 | 740880 | BD | 3 |
| CD19 | 1D3 | BUV661 | 612971 | BD | 3 |
| CD19 | 6D5 | Spark Blue 550 | 115566 | Biolegend | 1,2 |
| CD24 | M1/69 | AF647 | 101818 | Biolegend | 2 |
| CD24 | M1/69 | PE | 101808 | Biolegend | 1 |
| CD3 | 145-2C11 | PE-Cy5.5 | 35-0031-82 | Invitrogen | 1,2 |
| CD3 | 17A2 | AF532 | 58-0032-82 | Invitrogen | 3 |
| CD38 | 90 | APC-Cy7 | 102728 | Biolegend | 3 |
| CD4 | GK1.5 | BUV395 | 563790 | BD | 1 |
| CD4 | RM4-5 | BV 510 | 100559 | Biolegend | 3 |
| CD43 | S7 | BUV563 | 741238 | BD | 1 |
| CD44 | IM7 | BV 650 | 103049 | Biolegend | 3 |
| CD45 in vivo | 30-F11 | FITC | 103108 | Biolegend | 1-3 |
| CD45 in vivo | 30-F11 | BUV605 | 563053 | BD | 1-3 |
| CD45.2 | 104 | BV 605 | 109841 | Biolegend | 2,3 |
| CD45.2 | 104 | PerCP-Cy5.5 | 45-0454-82 | Invitrogen | 1 |
| CD62L | MEL-14 | BV 570 | 104433 | Biolegend | 3 |
| CD64 (FcγRI) | X54-5/7.1 | BV 711 | 139311 | Biolegend | 1,2 |
| CD69 | H1.2F3 | PE-Dazzle594 | 104536 | Biolegend | 3 |
| CD73 | TY/11.8 | PE | 127206 | Biolegend | 3 |
| CD8α | 53-6.7 | APC-R700 | 564983 | BD | 1,3 |
| CD8α | 53-6.7 | PerCP-Cy5.5 | 100734 | Biolegend | 2 |
| CD95 | SA367H8 | APC | 152604 | Biolegend | 3 |
| CXCR3 | CXCR3-173 | BUV395 | 745689 | BD | 2 |
| CXCR3 | CXCR3-173 | BUV563 | 741438 | BD | 3 |
| F4/80 | T45-2342 | APC-R700 | 565787 | BD | 2 |
| GL7 | GL7 | Pacific Blue | 144614 | Biolegend | 3 |
| IgD | 217-170 | BUV805 | 749299 | BD | 3 |
| IgM | R6-60.2 | BUV395 | 564025 | BD | 3 |
| KLRG1 | 2F1 | BV 750 | 746972 | BD | 3 |
| Ly-6C | HK1.4 | PE-Cy7 | 128018 | Biolegend | 1,2 |
| Ly-6G | 1A8 | BUV661 | 741587 | BD | 1,2 |
| MHC-II (I-A/I-E) | M5/114.15.2 | APC | 17-5321-82 | Invitrogen | 1 |
| MHC-II (I-A/I-E) | M5/114.15.2 | PE | 107608 | Biolegend | 2 |
| NK1.1 | PK136 | BV 785 | 108749 | Biolegend | 2 |
| Nrp1 | 3E12 | PE-Cy7 | 145212 | Biolegend | 3 |
| PD-L2 | TY25 | BUV496 | 741194 | BD | 3 |
| Siglec-F | E50-2440 | APC-Cy7 | 565527 | BD | 1,2 |
| TCRγ/δ | GL3 | AF647 | 118134 | Biolegend | 3 |
| Tetramer | N/A | BV 480 | Mkb-016 | Tetramer-Shop | 1,3 |
| Tetramer | N/A | BUV737 | Mkb-016 | Tetramer-Shop | 1,3 |
| Viability | N/A | Zombie NIR | 423106 | Biolegend | 1-3 |

**Supplementary Table S2.** List of predicted immunodominant SARS-CoV-2-S peptides binding to H2kb MHC class I molecules. The number in the peptide name also indicates peptide location within SARS-CoV-2-S protein.

| *Name* | *Sequence* | *Sub-pool for CD8^+^ T cell reactivation* | *Tetramer* |
| --- | --- | --- | --- |
| S-MHC1-133 | FQFCNDPFL | I-1 | - |
| S-MHC1-233 | INITRFQTL | I-2 | - |
| S-MHC1-234 | NITRFQTL | I-2 | - |
| S-MHC1-235 | ITRFQTLL | I-2 | - |
| S-MHC1-447 | GNYNYLYRL | I-3 | - |
| S-MHC1-449 | YNYLYRLF | I-3 | - |
| S-MHC1-539 | VNFNFNGL | I-4 | S-V8L |
| S-MHC1-539-2 | VNFNFNGLT | I-4 | S-V9T |
| S-MHC1-797 | FGGFNFSQI | I-5 | - |
| S-MHC1-798 | GGFNFSQI | I-5 | - |
| S-MHC1-798-2 | GGFNFSQIL | I-5 | - |
| S-MHC1-902 | MAYRFNGI | I-6 | - |
| S-MHC1-1005 | QTYVTQQL | I-7 | - |
| S-MHC2-345 | TRFASVYAWNRKRIS | I-MHC2 | - |
| S-MHC2-346 | RFASVYAWNRKRISN | I-MHC2 | - |
| S-MHC2-347 | FASVYAWNRKRISNC | I-MHC2 | - |
| S-MHC2-510 | VVVLSFELLHAPATV | I-MHC2 | - |
| S-MHC2-511 | VVLSFELLHAPATVC | I-MHC2 | - |
| S-MHC2-512 | VLSFELLHAPATVCG | I-MHC2 | - |
| S-MHC2-605 | SFGGVSVITPGTNTS | I-MHC2 | - |
| S-MHC2-606 | FGGVSVITPGTNTSN | I-MHC2 | - |
| S-MHC2-607 | GGVSVITPGTNTSNQ | I-MHC2 | - |
| S-MHC2-797 | AQVKQIYKTPPIKDF | I-MHC2 | - |
| S-MHC2-798 | QVKQIYKTPPIKDFG | I-MHC2 | - |
| S-MHC2-799 | VKQIYKTPPIKDFGG | I-MHC2 | - |
| S-MHC2-1047 | YHLMSFPQSAPHGVV | I-MHC2 | - |
| S-MHC2-1048 | HLMSFPQSAPHGVVF | I-MHC2 | - |
| S-MHC2-1049 | LMSFPQSAPHGVVFL | I-MHC2 | - |
| S-MHC2-1084 | AQEKNFTTAPAICHD | I-MHC2 | - |
| S-MHC2-1085 | QEKNFTTAPAICHDG | I-MHC2 | - |
| S-MHC2-1086 | EKNFTTAPAICHDGK | I-MHC2 | - |

**Supplementary Table S3.** List of antibodies used for immunofluorescence.

| Antigen | Clone | Fluorochrome | Species | Isotype | Supplier |
| --- | --- | --- | --- | --- | --- |
| B220 | RA3-3A1 | Cy5 | Rat | IgM | Homemade |
| CD3 | 145-2C11 | PE-Cy7 | Armenian Hamster | IgG | Invitrogen |
| CD21/35 | 7G6 | PE | Rat | IgG2bk | BD Pharmigen |

**Supplementary Table S4.** Serum cytokine concentrations at times indicated after i.n. boost determined by Ayoxxa’s **LUNARIS™ multiplex biomarker platform**. Data (n = 6-10) are expressed as mean group value (pg/ml) ± SD. * p <0.05 vs. buffer-treated mice at corresponding time point.

| Cytokine  (pg/ml) | buffer | | | sPB^8mu-7na^ | | | sPB^8mu-6na^ | | |
| --- | --- | --- | --- | --- | --- | --- | --- | --- | --- |
|  | **16 hpb** | **3 dpb** | **10 dpb** | **16 hpb** | **3 dpb** | **10 dpb** | **16 hpb** | **3 dpb** | **10 dpb** |
| INF-γ | 2.8±2.6 | 3.6±3.0 | 3.8±2.4 | 563.8±355.1* | 69.6±49.9* | 3.2±3.1 | 215.7±114.0* | 11.4±10.1 | 2.2±1.8 |
| IL-2 | 8.8±8.0 | 10.5±6.1 | 10.3±6.4 | 37.9±16.9* | 7.7±4.3 | 8.7±6.1 | 18.0±11.3 | 7.0±5.1 | 6.9±4.4 |
| IL-12p70 | 28.0±29.6 | 31.6±25.1 | 38.2±25.6 | 138.4±89.9* | 23.0±17.6 | 36.0±41.7 | 61.0±59.1 | 23.6±22.8 | 19.3±14.9 |
| IL-6 | 11.6±14.7 | 35.4±32.0 | 16.4±11.1 | 56.6±26.6* | 37.8±58.4 | 17.4±20.5 | 24.5±17.8 | 25.1±16.6 | 5.3±6.1* |
| TNF-α | 17.5±17.0 | 19.0±9.8 | 39.1±51.3 | 36.9±20.9 | 31.9±20.0 | 27.3±24.4 | 30.6±31.3 | 38.5±50.1 | 25.2±28.4 |
| IL-1β | 18.8±15.5 | 25.4±15.6 | 25.9±18.1 | 25.4±14.0 | 17.8±11.5 | 28.6±29.0 | 16.1±13.6 | 20.7±17.9 | 12.7±10.6 |
| IL-17 | 12.9±10.5 | 15.5±14.5 | 17.0±12.6 | 14.0±9.5 | 9.2±8.2 | 12.7±11.8 | 8.5±7.7 | 8.4±5.7 | 8.0±6.0 |
| IL-4 | 113.4±17.3 | 101.3±0.0 | 105.8±11.1 | 108.3±20.9 | 103.4±19.6 | 106.1±25.1 | 109.6±25.1 | 98.1±9.6 | 109.4±44.8 |
| IL-13 | 15.5±7.6 | 13.7±1.5 | 13.5±8.9 | 11.6±1.5 | 10.3±4.1 | 9.8±4.1 | 11.2±4.3 | 15.4±8.9 | 14.0±7.0 |
| IL-5 | 32.2±14.6 | 17.6±12.6 | 17.2±12.2 | 224.4±64.7* | 64.0±33.9* | 12.3±10.8 | 140.1±50.5* | 32.2±19.3 | 13.9±9.9 |
| IL-10 | 5.9±4.1 | 3.6±1.2 | 6.0±3.0 | 4.7±3.0 | 6.4±5.2 | 4.5±3.6 | 3.7±2.6 | 6.4±3.9 | 4.7±5.1 |
